# Marine-Origin Polysaccharides and Their Chemically Modified Derivatives as Sources of Advanced Biofunctional Materials for Biomedical Applications

**DOI:** 10.1021/acs.biomac.4c01682

**Published:** 2025-07-24

**Authors:** Vera Sousa, Luís P. G. Monteiro, Djenisa H. A. Rocha, João M. M. Rodrigues, João Borges, João F. Mano

**Affiliations:** CICECO—Aveiro Institute of Materials, Department of Chemistry, University of Aveiro, Campus Universitário de Santiago, 3810-193 Aveiro, Portugal

## Abstract

Marine polysaccharides are widely available sustainable renewable macromolecules, which have attracted considerable attention owing to their enhanced biocompatibility, biodegradability, noncytotoxic, nonimmunogenic properties, and close similarity to the native cellular microenvironment of tissues and organs. Herein, a comprehensive overview of the main sources and properties of most studied cationic, anionic, and neutral marine-origin polysaccharides, their main chemical functionalization strategies, as well as their processing into advanced biofunctional materials/devices is provided. Several recent examples are given on the bottom-up processing of marine-origin polysaccharide-based biomaterials in the form of nano-/microparticles and capsules, nanofibers, thin films, membranes, hydrogels, cryogels, and (bio)­inks to be used as high added-value antimicrobial coatings, adhesives, and wound dressings, or in food packaging, cosmetics, controlled drug delivery, *in vitro* disease modeling, or tissue engineering and regenerative medicine. The main challenges hampering the clinical translation and commercialization of most marine-origin polysaccharide-based biomaterials and devices, and future perspectives in the field are also discussed.

## Graphical abstract

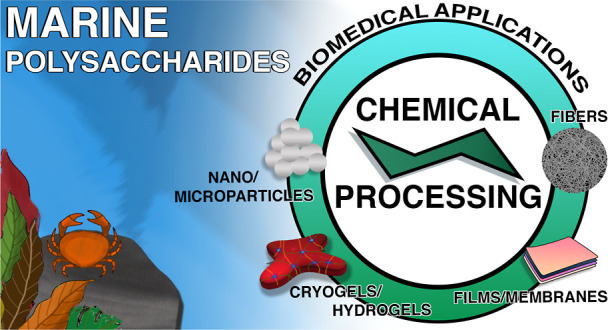

## 1 Introduction

The sea is an extraordinary and sustainable source of endless inspiration exhibiting countless diversity of natural compounds, which have been largely unexploited and widely discarded as leftovers of processed marine organisms due to their underestimated socioeconomic value and potential to be used in both biomedical and nonbiomedical arenas.^1,2^ In fact, the world capture of marine organisms, including aquaculture (mainly fish, molluscs, and crustaceans), amounts to 132 million tons,^3^ being more than 35% of the total weight discarded as waste, thus posing a major burden to human health and environment.^3^ Such abundant renewable marine biomass possesses tremendous potential as a ubiquitous source of bioactive molecules with a spectrum of structural diversity and chemical functionalities much vaster than the ones that can be achieved by chemical synthesis and standard chemical routes. Having this in mind and the pressing societal demand for a sustainable use of natural resources for the benefit of human well-being and the environment, the exploitation of the marine biodiversity is undoubtedly in the limelight.^4^ There is, indeed, the need to maximize the value of such marine biomass and byproducts as an unprecedented source of bioactive molecules for the development of high added-value biomaterials, with both socioeconomic and environmental benefits, for addressing numerous biomedical and biotechnological applications, thus enabling the sustainable exploitation of marine resources.

Among the diversity of natural molecules that can be extracted from the marine environment, including from algae, plants, and animals, marine carbohydrates have raised considerable interest owing to their very appealing features, including biocompatibility, biodegradability, noncytotoxicity, easy and wide availability, nonimmunogenicity, and low cost. In particular, marine polysaccharides have blossomed the attention of the scientific community as high added-value macromolecules for several applications spanning from cosmetics, nutraceuticals, pharmaceuticals, and food^5^ to biomedical and biotechnological fields.^6–12^ In addition, although the focus of this review is on marine polysaccharides, their degradation products, i.e., marine oligosaccharides mainly derived from algae, have gained considerable attention in biomedicine in recent years due to their better water solubility, bioavailability, and biological activity. For further information on the progress, potential and impact of marine oligosaccharides, including their role in the development of biomaterials for bioapplications, readers are referred to other recent reviews.^13–16^ Recently, our group published an overview of green methodologies for the extraction, chemical modification and processing of most commonly used marine-origin polysaccharides into diverse biomaterial-based devices for healthcare.^17^ In addition, although one can find several reviews in the literature that focus on either the main sources, extraction and purification methodologies,^17–23^ or on the chemical and structural modification of a selection of specific marine polysaccharides,^24–29^ mainly derived from algae or animals, to create a library of novel polysaccharide derivatives to meet specific applications, a systematic overview is lacking, thus limiting the full potential, versatility, and attractiveness imparted by the vast pool of available marine polysaccharides in bioapplications.

This review emphasizes the main sources and extraction methods of a wide range of marine polysaccharides obtained from algae, plants, and animals, their key physicochemical, structural, and biological properties, as well as the diversity of chemical routes to impart them with multiple functionalities and improved bioactivity (Figure 1). Moreover, an overview of the most used nano- and microtechnologies to process marine polysaccharides into innovative and advanced biofunctional materials in the form of hydrogels, cryogels, films, membranes, fibers, particles, or capsules is provided for fulfilling a wide array of biomedical and biotechnological applications. Finally, future perspectives and opportunities imparted by exploring new chemical modification strategies are also discussed, as well as the current challenges behind the clinical translation and commercialization of most marine-origin polysaccharide-based biomaterials/devices.

**Figure 1:**
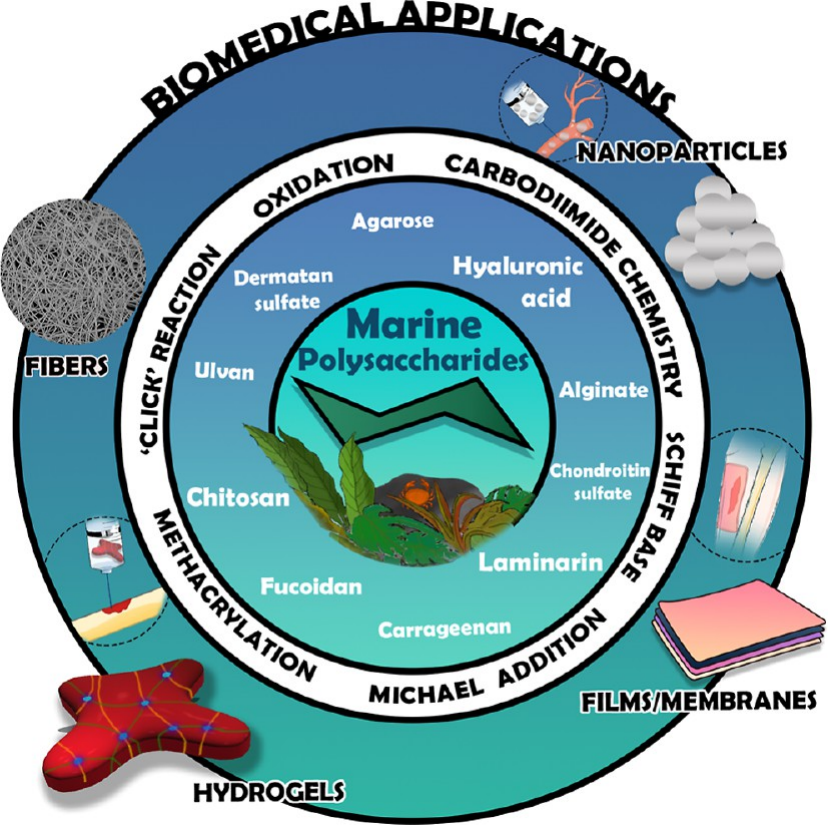
Schematic representation of key marine-derived polysaccharides—chitosan (CHT), agarose, laminarin (LAM), hyaluronic acid (HA), alginate (ALG), carrageenan, fucoidan, ulvan, dermatan sulfate (DS), and chondroitin sulfate (CS)—along with their diverse chemical modifications routes and versatile applications.

## 2 Marine Polysaccharides—Sources and Main Features

Over the last few decades, marine products have been gathering considerable attention as a major resource for pharmaceutical companies owing to their biocompatibility, biodegradability, noncytotoxicity, and nonimmunogenicity,^30^ as well as employability as active principles in a variety of therapeutic fields.^31^ Adding to this, marine polysaccharides can be easily chemically or physically tuned to obtain advanced biofunctional materials with interesting biomedical features. In this section, emphasis is given to the diverse marine sources and extraction methods of cationic (chitosan (CHT)), neutral (laminarin (LAM) and agarose), and anionic (alginate (ALG), hyaluronic acid (HA), dermatan sulfate (DS), and chondroitin sulfate (CS), ulvan, carrageenan, and fucoidan) polysaccharides derived from the marine environment. Although other negatively charged polysaccharides could have been covered, this review focuses on the most studied sulfated and carboxylated polysaccharides. For other polysaccharides, the reader is referred to other reviews.^32–34^

### 2.1 Polycationic

#### 2.1.1 Chitosan

Among the polysaccharides that can be extracted from the marine environment, chitin stands out because of its wide availability.^11^ Chitin, a β-(1,4)-linked linear polysaccharide consisting of *N*-acetyl-d-glucosamine repeating units (Figure 2A), is the second most abundant polysaccharide on earth after cellulose, and its most significant source is marine shellfish waste (e.g., shrimp, crab).^35,36^ Although chitin is abundant and denotes exceptional functional properties such as biocompatibility, biodegradability, and high mechanical strength, its bioapplication is widely hampered due to its insolubility in all regular solvents such as aqueous systems (mild acid or basic conditions) and organic solvents;^37^ consequently, chitin will not be addressed in this review. For a more in-depth discussion, readers are referred to other recent literature reviews.^38,39^ In the biomaterials field, great attention has been drawn to CHT, the main derivative of chitin.^11,35,40^ Due to its cationic nature, CHT is the most widely used and undoubtedly the most important polycationic marine polysaccharide. CHT can be obtained by the conversion of chitin through either enzymatic or chemical processes. The enzymatic conversion relies on the action of chitin-deacetylases, which catalyze the hydrolysis of the acetamido group. On the other hand, the chemical conversion is preferred due to its lower cost and suitability for mass production.^41^ Chemical deacetylation requires the treatment of chitin with hydroxides at high temperatures, usually above 80 °C,^42^ thus giving rise to CHT, a linear random copolymer with a molecular weight (MW) ranging from 50 to 2000 kDa, consisting of d-glucosamine and *N*-acetyl-d-glucosamine units linked by β-1,4 glycosidic bonds (Figure 2A). The ratio between these two functional units is considered as the degree of deacetylation.^43^ The chemical structure of CHT is unique due to the predominant presence of amino groups that can be ionized. In fact, when CHT is dissolved in an acidic environment, the amino groups become protonated and the polymer becomes cationic and, thus, susceptible to interact with oppositely charged molecules.^42,43^

**Figure 2:**
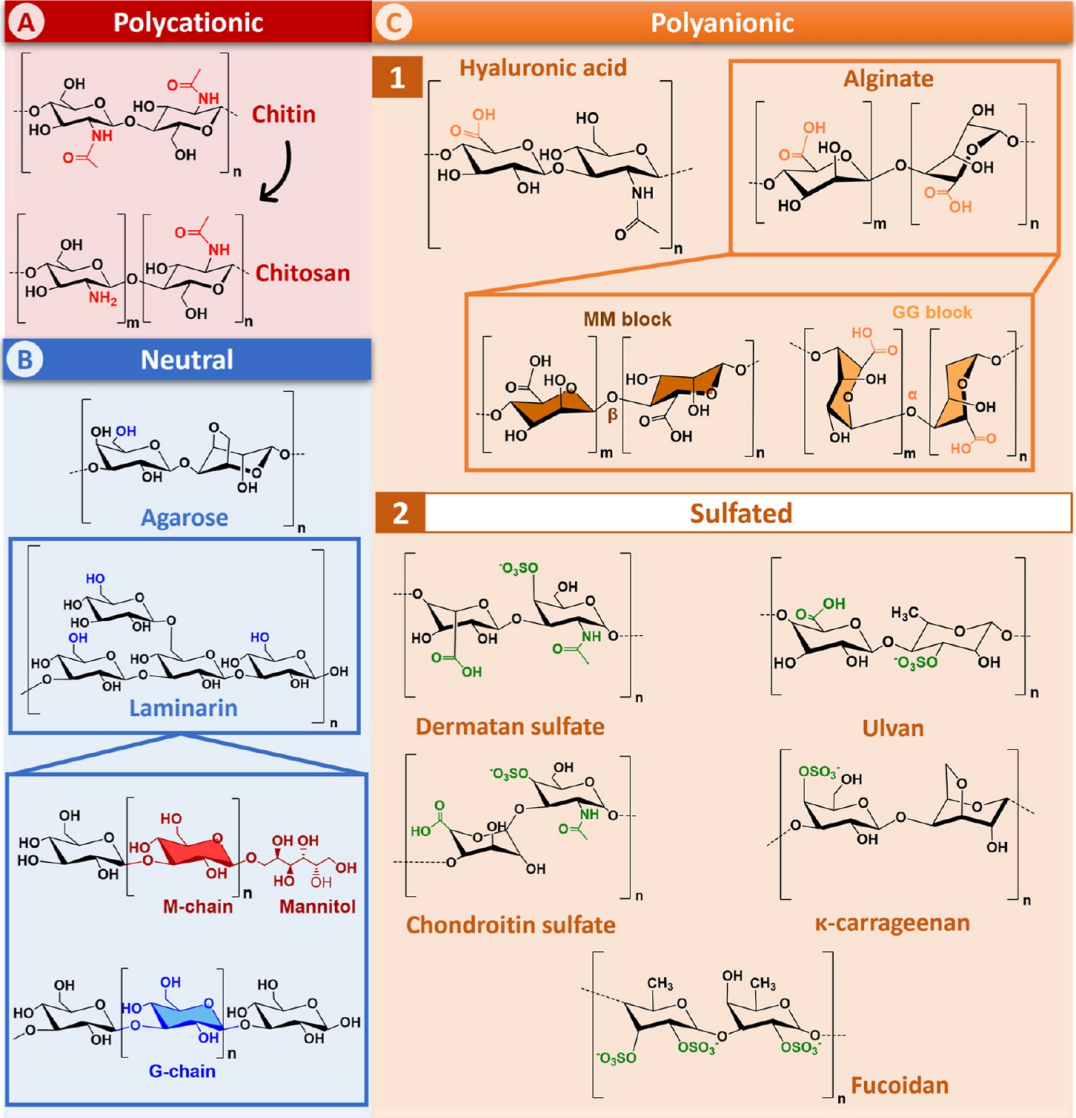
Chemical structures of the marine polysaccharides discussed in this review.

### 2.2 Neutral

#### 2.2.1 Agarose

Agarose is a linear polysaccharide extracted from red algae (e.g. *Gelidium* and *Gracilaria*) which consists of repeating units of agarobiose, a disaccharide encompassing 1,3-linked β-d-galactopyranose and 1,4-linked 3,6-anhydro-α-l-galactopyranose (Figure 2B). Agarose is the major component of agar (70%) and is obtained by removing the agar proteins from red algae’s extracts.^24^ The commonly used methods for the extraction and purification of agarose include ion exchange and agaropectin separation.^44^ An example of the latter was reported by Chew et al.^45^ who made use of polyethylene glycol (PEG) to precipitate agarose from *Gelidium amansii* red algae extracts. Furthermore, the extraction by ionic liquids has also proved to be a viable and “greener” approach, as the residues of the ionic liquid can be recovered and reused.^24^ In fact, some studies have reported the use of bioionic liquids. For example, choline laurate was found to be effective in the isolation of agarose with a concentration of only 4% (w/v) and an agarose extraction’s yield of ca. 14%. The authors also concluded that, when compared to commercially available agarose, the agarose extracted by this method retained the requisite purity essential for molecular biology applications, including gel electrophoresis.^46^ The MW of agarose is highly dependent on the extraction process; however, typically, the commercial agarose has a MW between 80 and 140 kDa.

#### 2.2.2 Laminarin

Initially isolated from *Laminariaceae* in the 19th century, LAM can be isolated from the cell wall of other brown seaweeds such as *Laminaria* sp., *Ascophyllum nodosum*, *Fucus vesiculosus*, and *Saccharina longicruris*, where it is used as a long-term carbon storage compound.^25,47^ LAM, also known as laminaran, is a polysaccharide consisting of (1,3)-β-d-glucopyranose residues with some 6-O-branching in the main chain and some β-(1,6)-intrachain linkages.^48^ In the LAM backbone, two types of polymer chains are presented: G-chains with glucose at the end and M-chains with mannitol as the terminal reducing end (Figure 2B). The MW of LAM is about 4–5 kDa, depending on the degree of polymerization, which is in the range of 20–25 glucose moieties.^48,49^ Moreover, it varies according to the extraction conditions. For instance, LAM extracted from *Laminaria saccharina* using hydrochloric acid has a higher MW when compared to that of LAM extracted with sulfuric acid.

Water solubility is a crucial factor in biomedical applications. In the case of LAM, its solubility in water is strongly influenced by its degree of branching. More specifically, highly branched LAM is soluble in both cold and hot water, while LAM with a low degree of branching is only soluble in hot water.^50^ Several conventional methods have been employed for LAM extraction, such as Soxhlet extraction,^51^ solid–liquid extraction,^48^ and liquid–liquid extraction.^52^ However, these methods rely on the use of harmful organic solvents, which turned attention to sustainable and more cost-effective modern extraction practices. Among these methods, enzyme-, ultrasound-, microwave-, and hydrothermal-assisted extraction, as well as supercritical fluid extraction have gained prominence as modern extraction techniques that not only enhance efficiency in terms of yield, time, and cost-effectiveness but also promote environmental sustainability by significantly reducing energy consumption.^52,53^ In fact, the extraction efficiency has been reported to be directly proportional to the temperature, being higher at high temperatures (50–90 °C) using water as a solvent.^54^ Although LAM has been extensively studied due to its potential biofunctional activities, it is nonadhesive, nonviscous, and does not form gels *per se*,^54^ thus turning its chemical functionalization of high demand.

### 2.3 Polyanionic

#### 2.3.1 Hyaluronic Acid

HA, also known as hyaluronan, is the only nonsulfated, anionic glycosaminoglycan consisting of β-1,4-d-glucuronic acid and β-1,3-*N*-acetylglucosamine repeating units (Figure 2C­(1)). The MW of HA is divided into three groups: high MW HA (HMW-HA), low MW HA (LMW-HA), and HA oligomers or oligosaccharides.^55^ In general, HMW-HA and LMW-HA have a MW ranging from 132 to 1700 kDa and 4.8–64 kDa, respectively.^56,57^ Commercially available HA is mainly obtained from animal tissues, typically rooster combs, or through microbial fermentation.^58^ However, it can also be obtained from various marine organisms, with cartilage being the most common source, especially from bivalve mollusks, the vitreous body of swordfish, tuna and sharks, and from the liver of stingrays.^59^ Due to its significant role in tissue engineering, HA remains a key polysaccharide in the development of biomaterials for regenerative applications and, as such, warrants discussion in this review. However, it is noteworthy that the majority of reports in the literature do not specify the origin of the HA used, which would enhance the reproducibility and enable a better comparison between studies, as variations in HA source can influence its molecular weight, purity, and biological interactions. Nevertheless, the main challenge in the purification of HA is the removal of the proteins of the proteoglycan matrix, which can be potentially allergenic. To minimize or even avoid this hurdle, several techniques have been employed, including precipitation in ethanol followed by protease-mediated hydrolysis,^60^ ultrafiltration-diafiltration, chromatography, and dialysis.^59^ Recently, enzymatic extraction of HA was explored using the vitreous humor of Nile tilapia.^61^ The extracted HA was found to have a MW of 37 kDa and exhibited superior thermal stability than commercially available HA extracted from bacterial sources (*Streptococcus equi*).^62^

#### 2.3.2 Alginate

ALG, also called algin or alginic acid, is one of the most exploited polysaccharides extracted from brown algae (mainly *Laminaria hyperborean*, *A. nodosum*, and *Macrocystis pyrifera*). ALG has a MW in the range of *ca*. 32–400 kDa and consists of 1,4-β-d-mannuronic acid (M) and 1,4-α-l-guluronic acid (G) monomers.^63^ The arrangement of the monomers can result in the formation of homogeneous MM and GG or heterogeneous MG/GM blocks—Figure 2C­(1).^64^ In fact, the arrangement of the M and G blocks dictates the final biological and physicochemical properties of ALG.^65^ Although ALG can be obtained from both algae and bacterial sources (mucoid strains of *Pseudomonas aeruginosa* or *Azotobacter vinelandii*), the commercially available ALG derives only from algae.^66^ Its extraction from brown algae encompasses six steps: (i) formaldehyde pretreatment of the dried algae, (ii) acid treatment, (iii) alkali extraction, (iv) bleaching, (v) precipitation, and (vi) drying. Its noteworthy that the alkali extraction step is crucial to increase the yield and physiochemical features of ALG. Most commonly, a sodium carbonate solution is used in this step to convert insoluble ALG into its water-soluble form, which is accumulated in the aqueous phase.^67,68^ In fact, Nesic and co-workers recently proposed a sustainable and cost-effective method to extract ALG from raw seaweed beach waste, specifically *Sargassum* algae.^69^ The authors initiated the process with a microwave-assisted treatment of the algae, followed by the use of three different extraction methods: a conventional procedure based on the demineralization process under acidic conditions, hot water, and an alkaline method. It was found that a combination of the latter with the microwave pretreatment method enabled the production of high-quality ALG, characterized by a high molecular weight and higher viscosity. In addition, although exhibiting lower extraction yields, the microwave pretreatment of the raw material allowed the extraction of ALG with higher MW (419–458 kDa) when compared to the one obtained by conventional methods (382 kDa). More importantly, this innovative approach reduced the extraction time by about 10-fold and required less volume of solvents.

#### 2.3.3 Sulfated

Sulfated polysaccharides, a subgroup within marine polysaccharides, exhibit a negative charge in solution. These polysaccharides are well-known for their significance in cellular communication, differentiation, and growth.^70,71^ Despite their heterogeneity, most sulfated polysaccharides share a common structure—a linear polysaccharide with a repeating unit of hexosamine and hexuronic acids linked by glycosidic bonds.^70^ Heparin, heparan sulfate, carrageenan, fucoidan, ulvan, CS, DS, and keratan sulfate are all well-known examples of sulfated marine polysaccharides.^72^ However, this review focuses on carrageenan, fucoidan, CS, DS, and ulvan.

##### 2.3.3.1 Carrageenan

Carrageenans encompass a vast family of sulfated polysaccharides extracted from red algae (*Eucheuma* and *Kappaphycus*), which are classified according to the position and number of the sulfate groups. Due to their gelation and thickening ability, carrageenans, particularly κ-carrageenan, have been widely used in food, cosmetic, and pharmaceutical industries.^32,73^ Their chemical structure consists in repeating disaccharide unit of (1,3)-linked β-d-galactose and (1,4)-linked α-d-galactose (Figure 2C­(2)) with an average MW ranging from 200 up to 800 kDa,^74^ where the galactose unit can be partially sulfonated at different positions. In fact, the specific sulfonation patterns determine their gelation ability.^75^ For example, κ- and ι-carrageenan, which have only one or two sulfate groups, respectively, can induce gelation in aqueous solutions, whereas λ-carrageenan, which has three sulfate groups, does not.^76^ κ-carrageenan is the most extracted type; however, ι- and λ-carrageenan are also available in nature. In fact, they can also be obtained by desulfonation of other types of carrageenan.^76^ Similarly to the above-mentioned polysaccharides, the extraction of carrageenan is carried out by hot water or alkali solutions. Even though hot water extraction has a higher yield, the gel strength of carrageenan is lower compared to alkali extraction.^32,77^ Due to their high energy and water costs, other extraction techniques have been proposed, such as ultrasonic or microwave-assisted extraction, which represent greener and less time-consuming approaches.^78^ In which regards to biological activities, carrageenan have been reported to be anticoagulant, antitumoral, antioxidant, and antiviral.^79,80^ These features along with the tunable viscosity and stimuli-responsiveness make carrageenan a promising candidate to develop novel biomaterials for biomedical application.

##### 2.3.3.2 Fucoidan, Chondroitin Sulfate, and Dermatan Sulfate

Fucoidan is another sulfated polysaccharide usually extracted from brown algae, sea urchins, and sea cucumbers. In contrast to the previously described marine polysaccharide, the structure of fucoidan is very source-dependent, and thus it does not have a typical structure. However, it is characterized by the presence of l-fucose (Figure 2C­(2)).^81,82^ For example, fucoidan extracted from *A. nodosum* mainly consists of repeating α-(1,3)-linked-l-fucopyranose residues with a sulfate group in the C-2 position, whereas when extracted from brown algae, fucoidan is composed of pure α-(1,3)-l-fucopyranose residues or alternating α-(1,3) and α-(1,4)-linked-l-fucopyranose with a sulfate group in the C-2 of the α-(1,3) sugar and at the C-2 or C-4 of the α-(1,4) units.^33,81,83^ Given the significant structural variability, its MW range is also very wide, going from 100 up to 5200 kDa.^33,84^ Regarding the extraction techniques from brown algae, the standard method involves hot acid treatment and/or CaCl_2_ to precipitate ALG;^83^ however, this usually results in undesirable contaminants (e.g., laminarin), MW reduction, and decreases the sulfate content; nonetheless, resorting to lower temperatures increases MW and sulfate content.^85^ For biological purposes, the MW should be below 30 kDa to enable cellular internalization. To obtain such low MW fragments, a depolymerization step is typically performed.^86^ However, in the field of biomaterials—where polysaccharides are rarely used in their native form—the MW is less critical. In fact, fucoidan exhibits several biologically relevant activities that contribute to the modulation of the biomaterial itself, including immuno-modulatory, antitumor, anti-inflammatory, anticoagulant, and hypoglycemic effects, among others.^87^

CS and DS share the same *N*-Acetylgalactosamine (GalNAc) unit but differ in the acidic monosaccharide moiety (Figure 2C­(2)).^88,89^ CS is primarily found in sea cucumbers, squids, and sharks, while DS can be isolated from ascidians^90^ and the skin of marine vertebrates such as crab, gray triggerfish, smooth hounds, and ray. However, a recent work proposed the extraction of CS from the jumbo squid cartilage by combining ultrasound-assisted enzymatic extraction and dialysis.^91^ The results showcased that, from a pool of three enzymes (alcalase, papain, and Protin NY100), alcalase presented the higher efficiency under highly controlled conditions (59.40 °C, 24.01 min, pH 8.25, and alcalase concentration of 3.60%). It was found that the combination of ultrasound-assisted enzymatic extraction and hollow fiber dialysis, widely used in hemodialysis, proved to be an effective and eco-friendly procedure to obtain pure CS. Similarly to HA, most reports on CS and DS do not specify the origin of the polysaccharide, which may hinder progress in the field by limiting the understanding of the structure–function relationship.

Regarding the sulfation position in CS, it can be categorized into ten classes. However, the most common CS types are CS-A and CS-C, being characterized by sulfonated C4 and C6 of the GalNAc unit, respectively. In fact, the type of CS and their MW vary according to the source. In particular, different CS types can be extracted from sharks, being the CS-A and CS-C the most prevalent ones (32% and 44%, respectively), with a MW ranging from 35 to 70 kDa.^92^ Likewise, the structure and disaccharide composition of DS vary depending on its origin, namely on the position of the sulfate groups in the DS backbone.^72^ Regarding its MW, DS isolated from rays, *Raja clavata* and *Raja radula*, has a MW of 31.2 kDa and 33 kDa, respectively.^93^ Furthermore, DS can be obtained through the incomplete epimerization of CS, where glucuronic acid is converted into iduronic acid.^89,94^

##### 2.3.3.3 Ulvan

Ulvan is a cell wall polysaccharide that accounts between 9 and 36% of the dry weight of the green algae *Ulva* and consists of repeating disaccharide units made up of α- and β-(1,4)-linked monosaccharides, mainly sulfated rhamnose, uronic acids (glucuronic acid and iduronic acid), and xylose (Figure 2C­(2)).^20,95^ The different types of ulvans can be classified into ulvanobiuronic acids (type A and B) and ulvanobioses (type U). Types A and B are the most common ones, both having a-l-rhamnose 3-sulfate unit. However, in type A, this unit is linked to a β-d-glucuronic acid unit, whereas in type B is linked to a α-l-iduronic acid moiety.^20^ The extraction of ulvan is predominantly conducted with hot water, typically maintained at temperatures between 80 and 90 °C. Enhanced extraction efficiency can be achieved through the incorporation of divalent cation chelating agents, such as ammonium oxalate.^20,27,96^ The extraction yields range from 8 to 29% of the algae dry weight and are intricately influenced by the source of the biomass, storage, pre-extraction conditions, extraction procedures and purification protocols.^66^ Recently, Thanh et al.^97^ reported for the first time the use of ultrasound-assisted extraction of ulvan from *Ulva lactuca* *.* After carefully adjusting the three main variables that can influence the extraction yield, namely, temperature, time and solvent ratio, an extraction yield of 22.5% was achieved with a MW of 231 kDa. The average MW of ulvan has been reported to be 53–320 kDa. Interestingly, different extraction temperatures lead to different MW. In particular, high extraction temperatures have been associated with the extraction of ulvan with high MW.^98^

## 3 Chemical Modification of Marine-Origin Polysaccharides and Their Processing into Advanced Biofunctional Materials

The chemical modification of the biopolymer structure can improve its water solubility, mechanical stability, bioactivity, and overall properties of the final biopolymer-based material. Their unique functionalities can be adjusted by coupling specific (bio)­functional groups, unlocking their potential for biomedical applications as novel functional biomaterials. The chemical modification of the ubiquitous hydroxyl groups present in the chemical structure of all polysaccharides has been in the limelight, although other functional groups have also been studied. In fact, the nucleophilic groups ranging from amines to alcohols, as well as the highly reactive carboxylic acid moieties, turn the polysaccharides into an ideal platform for a wide array of chemical modifications aiming to pursue biomaterials with improved properties and functions.^99^ In this review, an up-to-date overview of the main chemical strategies behind the functionalization of the aforementioned marine-origin polysaccharides in the last ten years is provided.

### 3.1 Chitosan

#### 3.1.1 Chemical Modifications

The presence of reactive functional groups in the CHT backbone, such as hydroxyl and amino groups, enables several straightforward chemical modifications for the construction of complex molecular architectures.^100^ The C-6 primary hydroxyl group and the C-2 amino group can lead to the formation of *O*-modified, *N*-modified, or *N*,*O*-modified CHT derivatives. Different modifications can occur within the CHT structure, with the most common being *N*,*O*-alkylation^101–103^ and *N*,*O*-acylation.^104–107^ Additionally, Schiff base reactions^108^ are frequently used in CHT due to the reaction between amine groups and aldehydes or ketones, forming imines. Moreover, the characteristic amine group of CHT can be converted to azide by a diazo transfer, obtaining N-azidated CHT, enabling “click” chemistry reactions.^109^ Less common modifications include phosphorylation,^110^ sulfonation,^111^ or xanthation.^112,113^

CHT possesses low solubility in both aqueous and organic solvents, thus hindering its conjugation with other synthetic organosoluble polymers. However, when biomedical applications are aimed at, the solubility of CHT in aqueous media is essential. To date, the most studied water-soluble chitosan derivatives are carboxymethyl CHT, obtained by the reaction of CHT with monochloroacetic acid; *N,N,N*-trimethyl CHT, obtained by the reaction of CHT with methyl iodide and, last, *N*-(2-hydroxy)­propyl-3-trimethylammonium CHT chloride, obtained by the reaction of CHT with 2-hydroxypropyl trimethylammonium (Scheme 1A).^114^ However, methyl iodine is highly toxic, imparting countless side effects. As such, considerable attention has been turned toward safer and greener reagents. Having this in mind, the chemical modification of amino and hydroxyl groups present in the d-glucosamine units with carboxymethyl moieties can be conducted to obtain a water-soluble carboxymethyl CHT derivative (CMC), whose water solubility across different pH environments can be tuned by playing with the degree of carboxymethylation.^115^ The carboxymethylation reaction can occur at the (i) primary hydroxyl groups, (ii) amino groups, or (iii) both hydroxyl and amino groups (Scheme 1B). It is well-known that the free amino groups of the d-glucosamine units of CHT are more reactive than the primary hydroxyl groups toward electrophile attack.^116^ With the same purpose, another chemical route to obtain water-soluble CHT derivatives involves reacting CHT with glycidyltrimethylammonium chloride (GTMAC) in water at 80 °C for 48 h (Scheme 1A).^117–119^ Moreover, the functionalization of CHT with alkyne-PEG spacers has been reported to significantly enhance its solubility in aqueous media. To achieve this, the authors first developed a mesylate salt of CHT, exhibiting improved solubility in both water and dimethyl sulfoxide (DMSO), followed by the activation of the alkyne-PEG-acid group using *N*-ethyl-*Ń*-(3-(dimethylamino)­propyl)­carbodiimide (EDC) and 1-hydroxybenzotriazole (HOBt) (EDC/HOBt) as activating agents. While the PEG chains improve CHT solubility, the alkyne-reactive terminal group facilitates further coupling via copper-catalyzed azide–alkyne cycloaddition (CuAAC) with azide-containing molecules.^120^ Additionally, CuAAC has also been successfully employed for the tethering of antimicrobial peptides (AMP), namely, Dhvar5, onto the CHT backbone.^121^ The same antimicrobial peptide was also conjugated to CHT via thiol-norbornene chemistry, yielding a higher conjugation efficiency. Briefly, this AMP-conjugated CHT was synthesized using a two-step approach: first, CHT was modified with carbic anhydride in a cosolvent system consisting of 0.1 M acetic acid in water and *N*,*N*-dimethylformamide (DMF); then, it was conjugated to a cysteine-bearing Dhvar5 peptide via UV-triggered thiol–ene click chemistry.^122^ Besides the successful introduction of bioactive entities, CuAAC can also be seen as a powerful tool for developing a new library of materials with intriguing properties. For instance, the synthesis of a pyranoflavylium-functionalized CHT derivative, aiming to develop a pH-sensitive chromogenic membrane, was accomplished using CuAAC between alkyne-modified CHT and azide-pyranoflavylium.^123^ Despite the regioselective nature of this chemistry, a catalyst was needed, such as TBTA (tris­((1-benzyl-4-triazolyl)­methyl)­amine). “Click” chemistry has not been limited to grafting small molecules or peptides onto the CHT backbone; it has also been employed as a cross-linking strategy with biomacromolecules. A thiol–maleimide “click” reaction was recently employed to cross-link thiol-modified carboxymethyl CHT and maleimide-oxidized sodium ALG.^124^ CMC was functionalized with thiol groups via a one-step amidation reaction, and upon mixing the two polysaccharides, a Michael addition “click” reaction occurs between the thiol groups of CMC derivative and the maleimide groups of ALG. On the other hand, the bromoacetylation reaction of CHT (Scheme 1C) has also been reported as a viable method to enhance the CHT reactivity with bioactive molecules containing amino groups.^116,125,126^

**Scheme 1:**
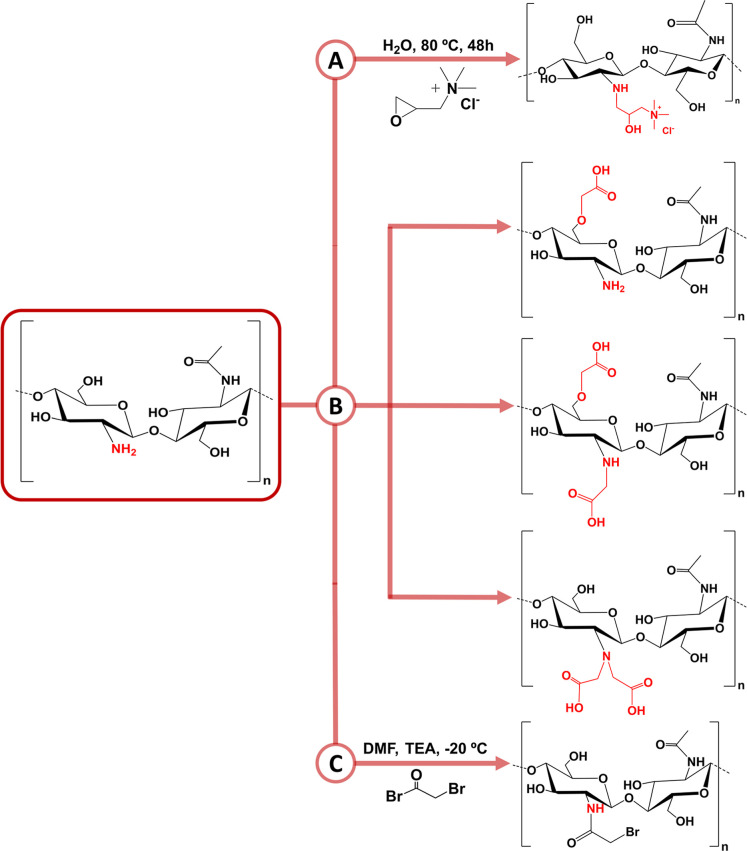
(A) Synthesis of Quaternized CHT (CHT–GTMAC); (B) Carboxymethylation and Its Reaction Sites; and (C) Bromoacetylation of CHT

There is a growing interest in the biological application of derivatives obtained from the modification of CHT through Schiff base reaction, specifically with heterocycles containing nitrogen, sulfur, and oxygen groups.^127–129^ For instance, the functionalization of CHT with aromatic aldehydes, namely, *p*-nitrobenzaldehyde (NBA) groups, across the condensation of the aldehyde groups of NBA with the aminated CHT (Scheme 2) improved significantly the antibacterial and antioxidant properties of the native polysaccharide.^128^ However, this approach is not time-efficient, as it involves multiple steps. A recent study reported the synthesis of Schiff base CHT derivatives, with both antimicrobial and antifungal activities, using simpler and more cost-effective methods, namely: (i) a one-pot high-temperature procedure and (ii) an ultrasound-assisted synthesis, with the later leading to higher yields.^129^

**Scheme 2:**
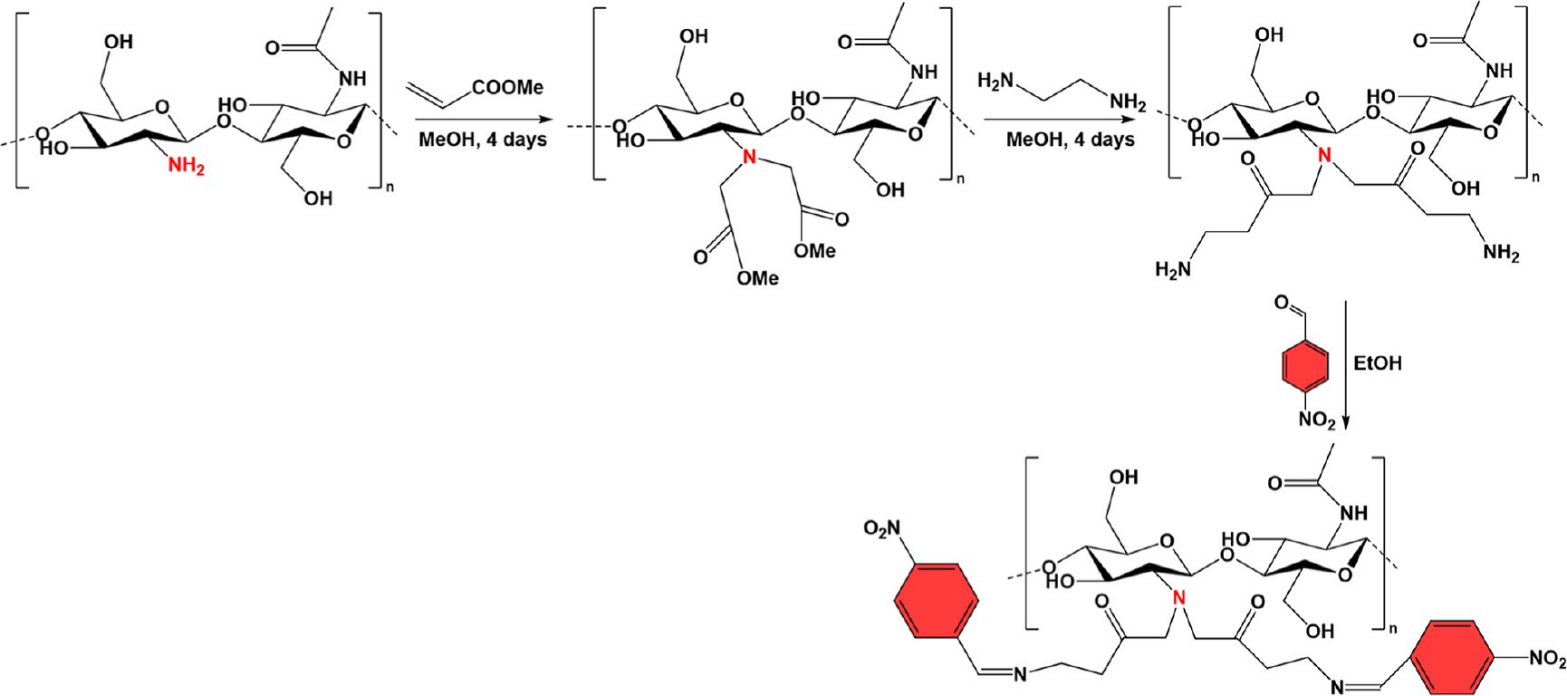
Modification of CHT with NBA Groups via a Schiff Base Reaction

Another chemical route that takes advantage of the amino groups present in the CHT backbone is the *N*-ethyl-*Ń*-(3-(dimethylamino)­propyl)­carbodiimide/*N*-hydroxysuccinimide (EDC/NHS) carbodiimide coupling reaction, which opens new avenues toward advanced (bio)­materials. For example, a thermoresponsive hydrogel was developed via EDC/NHS chemistry by combining CHT with a recombinant human collagen-peptide (RHC).^130^ It was found that the coupling reaction rate increases with the molar ratio of EDC (relative to the –COOH of RHC). The same trend was observed with the reaction time, with a 24 h time showing a higher coupling efficiency. Similarly, the optimization of the PEGylation of CHT through EDC/NHS chemistry was also studied, resulting in a degree of substitution of 24% when the reaction was conducted at pH 5 and using a molar ration of 3.5:1:1:10 (NH_2_/COOH/NHS/EDC).^131^ The same chemistry was employed in CHT/gelatin films, aiming to improve their mechanical properties by fine-tuning the CHT/gelatin ratio, which can also affect other features such as water absorption, swelling, and transparency—crucial considerations for advanced biomedical applications, particularly in corneal tissue regeneration.^132^

The Michael addition reaction, which involves the interaction between a nucleophile and an unsaturated carbonyl compound, has demonstrated its efficacy under physiological conditions without the need for toxic reagents or the formation of undesirable side products. Taking this into account, Oh et al.^133^ showcased the synthesis of a thermosensitive *N*-hexanoyl glycol CHT that was further modified with glycidyl methacrylate and 3-mercaptopropionic acid to yield methacrylated hexanoyl glycol CHT (M-HGCHT) and thiolated hexanoyl glycol CHT (SH-HGCHT), respectively (Scheme 3). The mixture of the two derivatives, M-HGCHT and SH-HGCHT, ensures the retention of the thermogelation properties conferred by the hexanoyl glycerol pendent groups while also maintaining their reactive functionalities for chemical cross-linking at 37 °C by the Michael addition reaction.

**Scheme 3:**
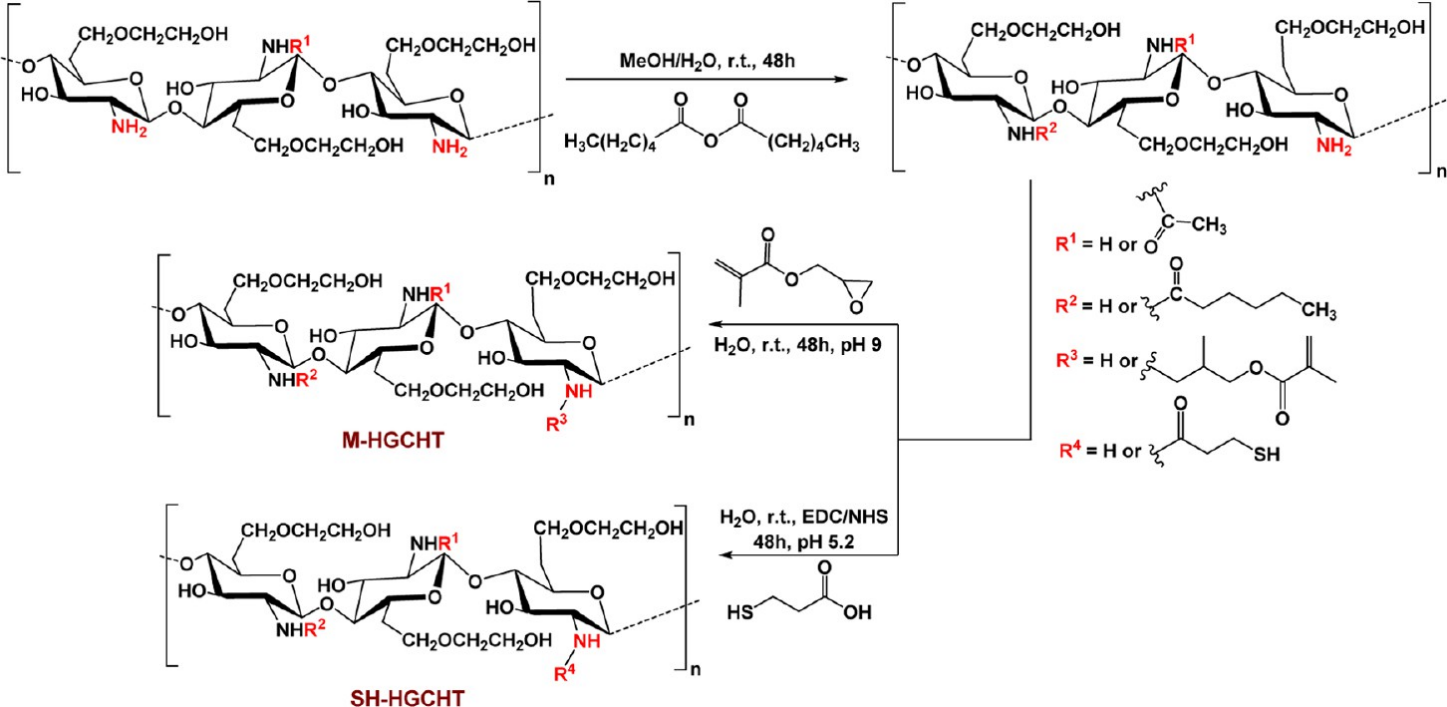
Synthesis of Methacrylated Hexanoyl Glycol CHT (M-HGCHT) and Thiolated Hexanoyl Glycol CHT (SH-HGCHT)

#### 3.1.2 Biomedical Applications

##### 3.1.2.1 Soluble Derivatives

As previously mentioned, the insolubility of native CHT in aqueous media limits its application in the biomedical field. Driven by this bottleneck, a study conducted by Rizwan et al. reported an organosoluble CHT derivative that, besides showing enhanced antibacterial activity when compared with the native CHT, could enable the production of films and nanofibers when combined with PCL.^134^ With the goal of overcoming the insolubility of CHT in physiological conditions while preserving the biological activity of bioactive molecules, a recent report details the synthesis of *N*-(2-hydroxypropyl)-3-trimethylammonium chitosan chloride (HTCC), a water-soluble quaternized CHT derivative, and its use as a building block, together with oppositely charged ALG, to engineer HTCC/ALG free-standing multilayered membranes for controlled drug release.^119^ The authors demonstrated the feasibility of achieving a more sustained release overtime by incorporating BSA as an intrinsic building block of the layer-by-layer membrane (Figure 3A, left panel). On the other hand, when BSA was applied as an outer layer (Figure 3A, right panel), the membranes exhibited a burst release within the first 4 h (Figure 3B). In addition, the mechanical properties of the free-standing membranes built with HTCC were compared with those of the membranes built with native CHT (Figure 3C), with the former ones revealing a higher tensile strength, irrespectively of the pH of the assembly process.

**Figure 3:**
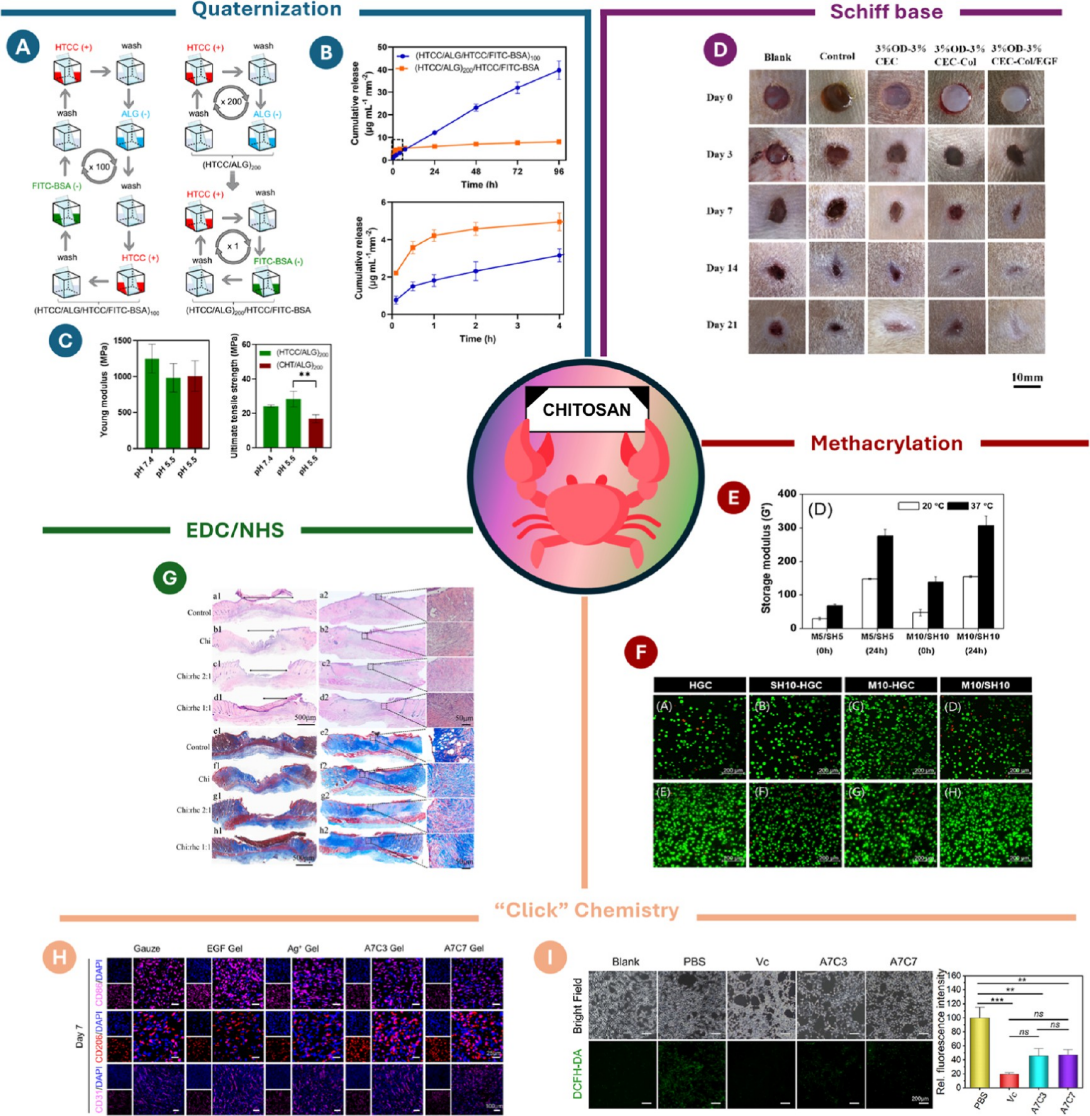
(A) Scheme of the two setups used to assemble the two types of free-standing multilayered membranes, either using BSA as an intrinsic building block (left panel) or using BSA as an outer layer (right panel); (B) cumulative release profile of FITC-BSA in PBS at pH 7.4 from the two types of free-standing membranes; (C) mechanical properties of the membranes, comprising the quaternized CHT or native CHT. Reproduced from,^119^ with permission from Royal Society of Chemistry; (D) images depicting full-thickness wounds treated with different formulations, including the control group (commercial dressing). Reprinted with permission from Elsevier;^135^ (E) effect of the temperature on the mechanical properties of the CHT thermogels; (F) Live/dead images of encapsulated cells within the CHT-based thermogels after 1 day of culture. Reprinted with permission from Elsevier;^133^ (G) histological analysis of wound sections collected from rats before (above, left) and after 14 days of treatment (above, right). Masson trichome staining of wounds after 14 (bottom, left) and 21 days (bottom, right) of treatment. Reprinted with permission from Elsevier.^130^ (H) Wound healing morphology at different time points with gauze, EGF gel, and Ag^+^ gel as controls. (I) Fluorescence staining showing the hydrogel’s effect on scavenging intracellular ROS (left) and relative fluorescence intensity of intracellular ROS after hydrogel treatment (right). Reprinted with permission from Elsevier.^124^

##### 3.1.2.2 Schiff Base

The CHT-derived Schiff base materials have proven to be promising candidates for a vast array of biomedical applications due to their biodegradability, biocompatibility, metal-binding capability and antibacterial properties.^127^ For instance, the combination of CHT with *p*-nitrobenzaldehyde (NBA) results in a derivative that exhibits enhanced antibacterial, antioxidant, and antibiofilm properties when compared to native CHT. Furthermore, the CHT derivative supported cell proliferation without significant hemolysis.^128^ With the same purpose and by resorting to a more straightforward approach, a novel Schiff base CHT derivative—obtained via ultrasound-assisted reaction of CHT with 4-oxo-4H-chromene-3-carbaldehyde—was recently developed. This derivative demonstrated notable antimicrobial activity against both Gram-positive and Gram-negative bacteria as well as antifungal properties against *Candida albicans* and *Candida tropicalis*.^129^ Taking advantage of the same coupling chemistry and by combining collagen with epidermal growth factor (EGF), an *in situ*-forming dextran/CHT hydrogel was developed by Hu and co-workers,^135^ denoting improved mechanical strength provided by collagen and enhanced biocompatibility granted by EGF. Furthermore, a significant acceleration in the wound healing process was denoted, with a remarkable 86% closure being achieved within 14 days (Figure 3D).

##### 3.1.2.3 Methacrylation

The high content of amino groups in the CHT backbone makes this cationic marine polysaccharide highly susceptible to various chemical modifications, thus enabling its interaction with diverse types of molecules, as depicted in Table 1. There are several reports on the methacrylation of CHT through different synthetic routes to obtain biomaterials with promising features.^136,137^ For instance, a recent study reported the development of a method to enhance the coupling and gel formation efficiency in CHT methacrylation reaction by resorting to EDC/NHS as a cross-linking agent.^138^ The authors aimed to evaluate the cell morphology of osteoblast-like MG63 cells in the CHT hydrogels; however, due to the low solubility of the methacrylated CHT, diluted PBS/diluted culture medium was used to dissolve the methacrylated CHT enabling the examination of the hydrogels’ biological performance. Furthermore, the rapid formation of the hydrogel ensured suitable cellular tolerance during brief exposure to the diluted culture medium. This work showcased that the cells could remain viable even in a relatively diluted culture medium, which acts as a source of inspiration for other scientists to design, construct, and study similar hydrogels.

**Table 1:**
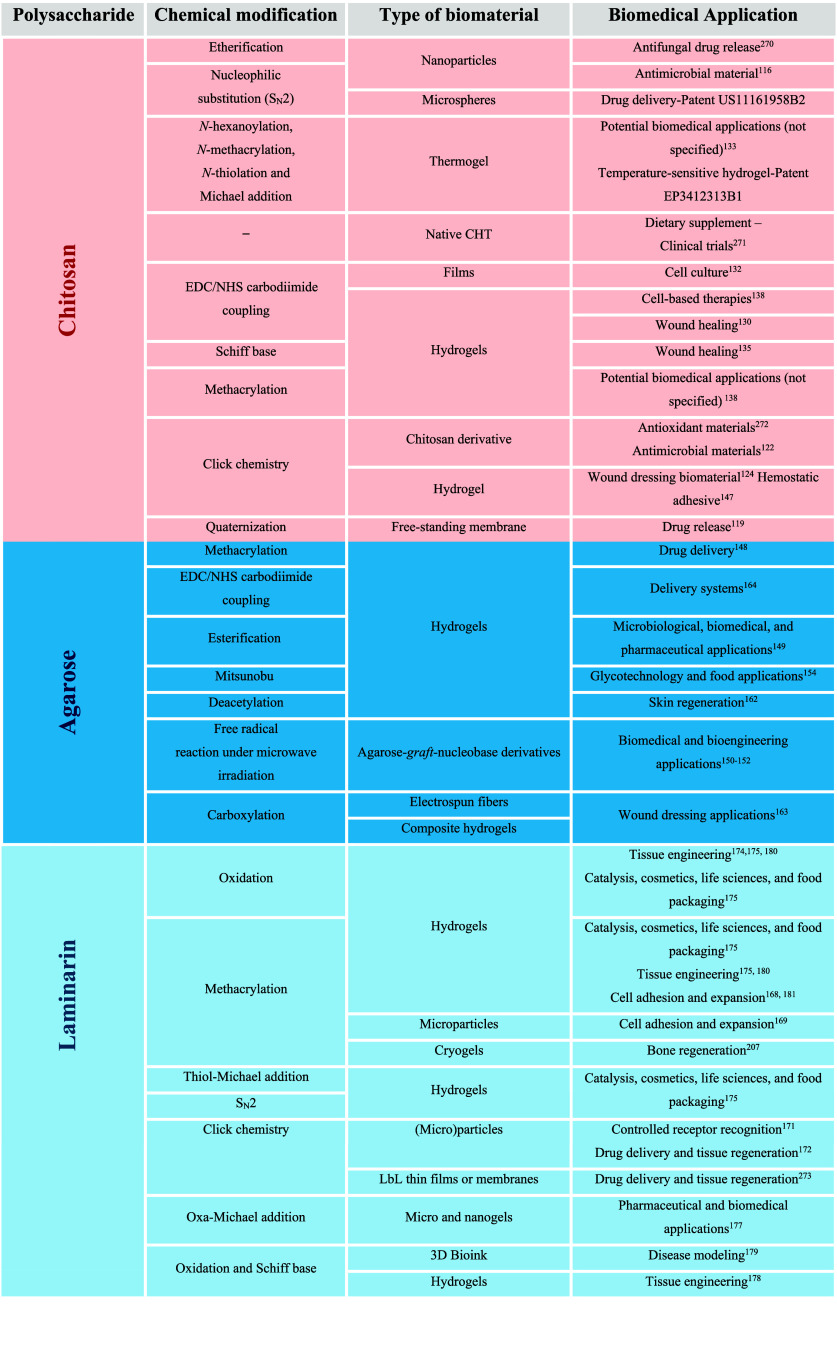

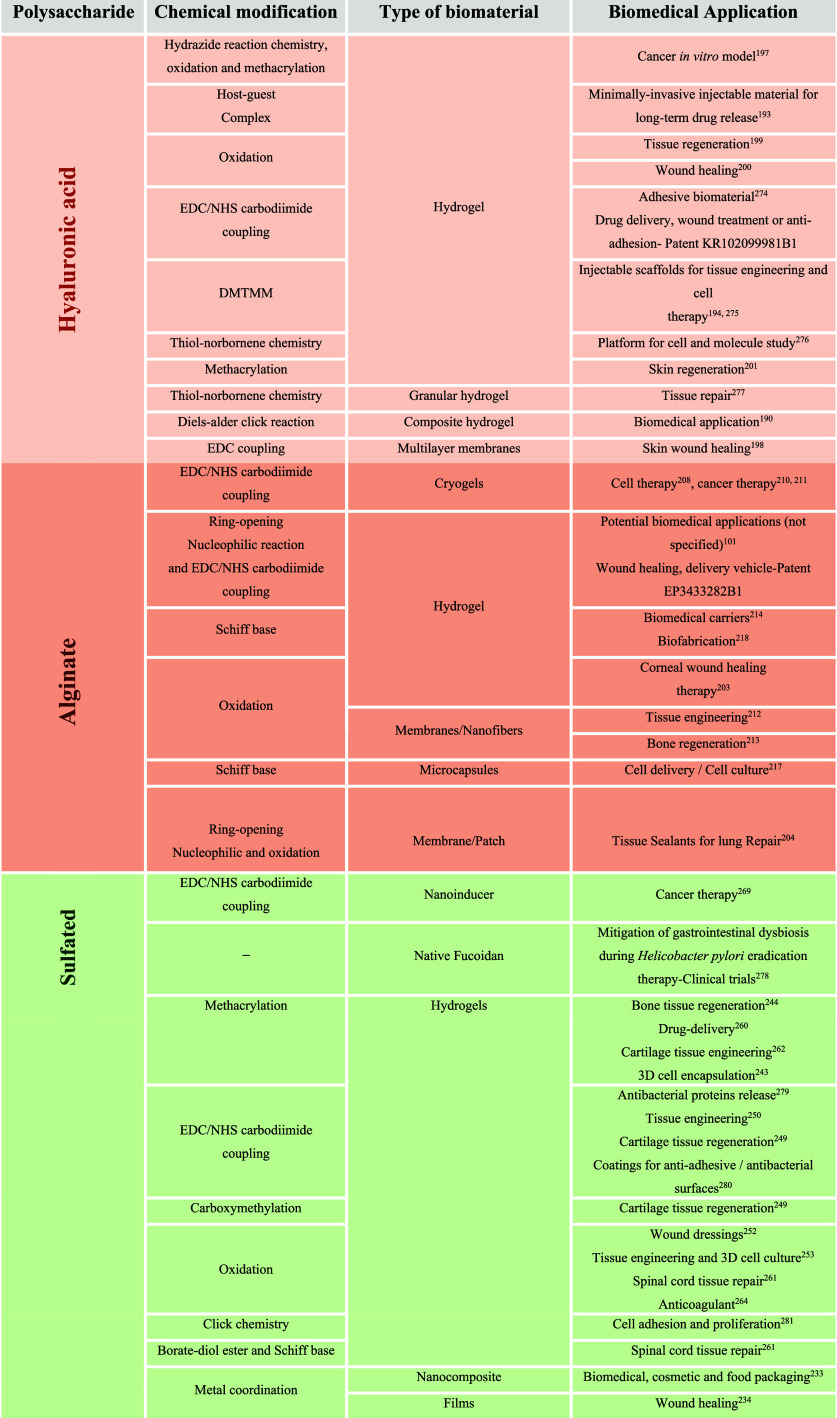
Marine-Derived Polysaccharides and Their Chemical Modification Routes to Obtain Advanced Biomaterials for Biomedical Applications

In many cases, to achieve biomaterials with enhanced properties and multifunctionalities, more than one chemical modification is needed. In this regard, it was reported the development of an *in situ*-forming dual-cross-linked CHT thermogel by mixing M-HGCHT and SH-HGCHT that undergoes thermogelation at 37 °C via hydrophobic interactions (physical cross-linking).^133^ Then the chemical cross-linking occurs through the Michael addition reaction between the methacrylated and thiol moieties (Figure 3E). *In vitro* experiments revealed that, despite the chemical cross-linking process, this novel *in situ* thermogel was not cytotoxic (Figure 3F), suggesting that it might be a safer and alternative solution to UV-based *in situ* hydrogels where cell encapsulation is a major hurdle.

##### 3.1.2.4 EDC/NHS

The EDC/NHS carbodiimide coupling chemistry takes advantage of the free amino groups that are present in CHT backbone either by enabling the coupling of bioactive agents^107,108^ or by acting as a cross-linking agent.^130,132^ As an example of this latter group, Shahin et al.^132^ resorted to the EDC/NHS chemistry to cross-link CHT/gelatin films for corneal epithelial cell culture aiming for cornea tissue regeneration. First, CHT and gelatin were mixed in specific ratios, dried, and mixed in a solution containing EDC and NHS. Lastly, this mixture was transferred to a mold that mimics the curvature of contact lens. It was found that increasing the CHT concentration improved the transparency, water permeability, degradability, cell viability, and proliferation. Thermoresponsive hydrogels prepared through the conjugation of recombinant collagen-peptide (RHC) and CHT through EDC/NHS coupling chemistry have been used for wound healing.^130^ The thermoresponsive hydrogels exhibited high mechanical strength and superior cell viability when compared with hydrogels incorporating only CHT. *In vivo* studies demonstrated that these hydrogels facilitated cell infiltration, induced blood vessel formation, and, ultimately, accelerated wound healing (Figure 3G). Notably, the control group (rats not subjected to treatment with the hydrogels) exhibited disorganized and sparse collagen deposition when compared with the RHC–CHT hydrogels (Figure 3G).

##### 3.1.2.5 “Click” Chemistry

“Click” chemistry has emerged as an efficient strategy for covalently grafting both small and large (bio)­molecules. When applied to CHT, it has been widely implemented either as a tool to covalently bind small molecules or as a cross-linking strategy.^139–142^ For example, an antimicrobial peptide was grafted onto CHT by photoclick chemistry to increase the antimicrobial activity of CHT derivative.^122^ The authors concluded that CHT functionalized with the antimicrobial peptide exhibited an impressive 35% reduction in bacterial adhesion compared to native CHT against *Staphylococcus epidermidis*. When tested against *P. aeruginosa*, the synthesized CHT derivative exhibited significant bacterial death, leading to over twice the bacterial mortality observed with native CHT. However, while the peptide-CHT conjugate displayed good antimicrobial activity, it did not show a significant reduction in bacterial viability compared to the peptide *per se*. Furthermore, the authors reported that the antimicrobial effects were also influenced by the limited degree of peptide grafting within the CHT.

This type of chemistry can also be used as a cross-linking method,^143–145^ as demonstrated by Lei et al.^124^ In this work, the authors proposed a hydrogel based on the Thiol-Michael addition “click” reaction between maleimide-ALG and thiol modified CHT to serve as a wound dressing biomaterial. The formulated hydrogels exhibited tunable gelation time, with a short gelation time (3.6 s) for the highest concentration of polysaccharides; however, this formulation also presented the lowest ultimate strain. Regarding the immunomodulatory effects, fluorescence assays revealed that the hydrogels promoted superior levels of M2-type macrophage polarization when compared with gauze and EGF gel (controls), as seen by the upregulation of CD206, a marker associated with anti-inflammatory M2 macrophages, and the downregulation of CD86, which is typically expressed in pro-inflammatory M1 macrophages (Figure 3H). Given the presence of reducing groups in the hydrogel network, the hydrogels also exhibit the ability to scavenge reactive oxygen species (ROS) in oxidative stress environments, as concluded by ROS fluorescence staining in a NIH-3 T3 oxidative stress model (Figure 3I). Both hydrogel formulations exhibited lower fluorescence levels (lower ROS concentration) than PBS and comparable levels with vitamin C (positive control). Lastly, *in vivo* experiments confirmed that the hydrogels accelerate wound healing in infected sites, while reducing scar formation. Despite the numerous advantages of “click” chemistry, the reliance on copper as a catalyst, particularly for CuAAC, poses a significant limitation for biomedical applications due to potential cytotoxicity and residual contamination. To address this limitation, copper-free “click” reactions, such as strain-promoted azide–alkyne cycloaddition (SPAAC), have been investigated as viable alternatives, either by functionalizing CHT with azide^146^ or alkyne groups.^147^ An example of the latter is a double-network CHT hydrogel based on dibenzocyclooctyne (DBCO)-functionalized CHT and four-arm PEG tetrazide, as recently reported.^147^ CHT was also modified to bear catechol groups for enhanced adhesiveness. Upon SPAAC, the hydrogel’s compressive strength increased almost 6-fold when compared to their counterpart lacking the synthetic polymer. Further assays revealed that the designed hydrogels outperform commercially available fibrin glue in terms of adhesive strength and hemostatic ability. Lastly, the hydrogels display the ability to promote collagen deposition, skin regeneration, and revascularization of the damaged tissue.

### 3.2 Agarose

#### 3.2.1 Chemical Modifications

Novel agarose derivatives can be obtained by modifying the primary alcohol group at C-6 of the (1,3) linked β-d-galactose moiety via esterification, amination, amidation, and C–N bond formation. The example presented in Scheme 4A shows the formation of methacrylate agarose using methacrylic anhydride, DMSO as a solvent, and 4-dimethylaminopyridine (DMAP) as a weak base.^148^ In a different approach, the conjugation of agarose with amino acids has been also described using a similar method under microwave irradiation.^149^ This modification allowed the formation of hydrogels with potential applications in microbiology, biomedical, and pharmaceuticals fields.

**Scheme 4:**
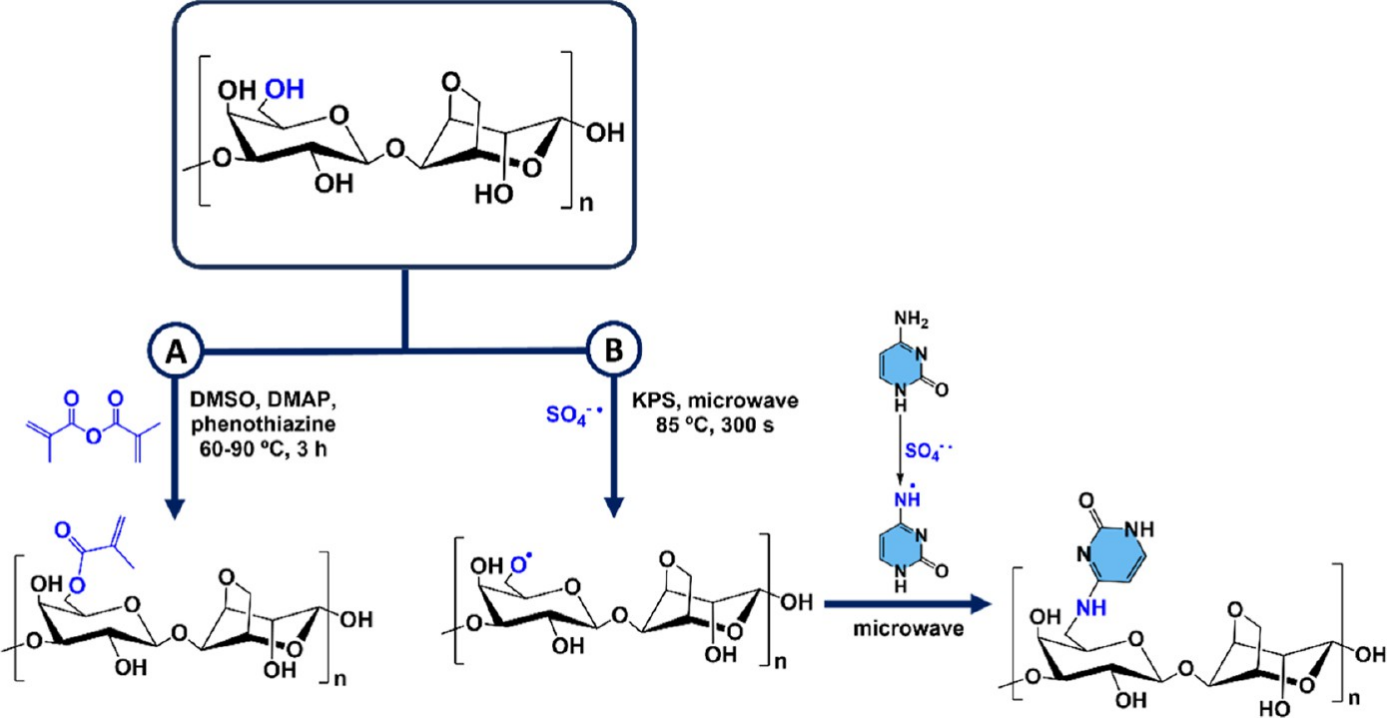
Methacrylation of Agarose (A) and Synthesis of Agarose-*graft*-nucleobase (B)

The C–N bond formation in the agarose structure could be achieved by free radical mechanism, where primarily the sulfate anion radical is formed from the potassium persulfate (KPS) under microwave irradiation. The radical ions located on C-6 of agarose and the nucleobase radicals (cytosine, adenine, and guanine) on the primary amine react with each other. This reaction resulted in the formation of agarose-*graft*-nucleobase, as corroborated by previous studies^150–152^ (Scheme 4B). On the other hand, sodium saccharate-agarose derivatives can also be obtained through *in situ* C–N bond formation by a two-step reaction. First, the substitution of the primary OH group by iodine groups at C-6 was performed. Then, the iodide group was replaced by saccharate sodium salt, yielding the desired conjugate.^153^

Amination and/or amidation of agarose are expected to be valuable modifications at the C-6 carbon position, aiming to obtain derivatives suitable for biomedical and pharmaceutical applications. The 6-aminoagarose was synthesized by Mitsunobu reaction involving the reaction of agarose with phthalimide in the presence of diisopropyl azodicarboxylate/triphenylphosphine (DIAD/TPP), followed by hydrazinolysis (Scheme 5A).^154^ The Staudinger reaction was also described as an alternative method to obtain the same derivative.^155^ The coupling reaction of 6-aminoagarose with different carboxylic acid compounds using EDC/NHS coupling afforded modified polysaccharides with strong fluorescent properties.^154^

**Scheme 5:**
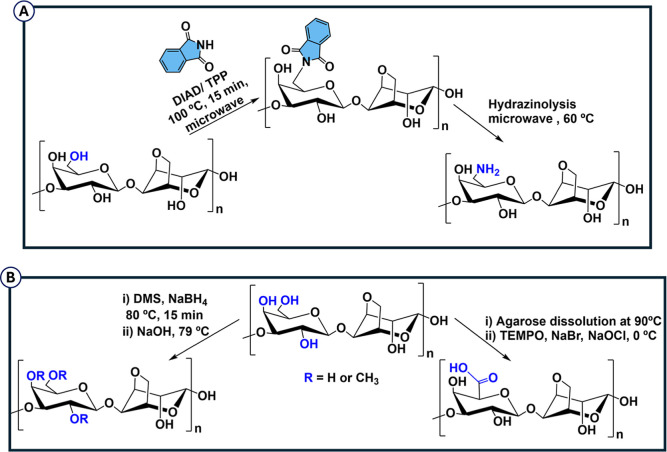
(A) Synthesis of 6-Aminoagarose by Mitsunobu Reaction; (B) Chemical Functionalization of Agarose with Methyl and Carboxylate Terminal Groups

Methylation of agarose with dimethyl sulfide (DMS) is a safe and simple method for the production of methylated agarose with potential application in biomedical, food and pharmaceutical areas.^156^ The carboxylation of the primary alcohol of agarose using (2,2,6,6-tetramethylpiperidin-1-yl)­oxyl (TEMPO) enables the production of agarose with varying degrees of carboxylation (28, 60 and 93%), imparting it with antimicrobial properties^157^ (Scheme 5B). However, agarose, in the native state, i.e. without any chemical modifications, can form a gel upon heating and cooling below the upper critical solution temperature.^158^ This capacity to produce functional biomaterials without chemical modification is both a more cost-effective and a less time-consuming process.^159–161^

#### 3.2.2 Biomedical Applications

##### 3.2.2.1 Carboxylation

Agarose, a commonly used marine polysaccharide for gel electrophoresis, has been also explored as a biomaterial to obtain 3D matrices for several biomedical applications, including drug delivery,^148^ skin regeneration,^162^ and wound dressing applications.^163^ There are several chemical routes for obtaining different multifunctional agarose scaffolds, as shown in Table 1. For instance, by employing the carboxylation reaction, carboxylated agarose-based electrospun fibers were developed (Figure 4A) exhibiting excellent antimicrobial properties against common wound pathogens, including *Staphylococcus aureus* and *P. aeruginosa*.^157^ Using the same agarose derivative, Ninan and co-workers^163^ developed a pH-sensitive composite hydrogel for wound healing via Zn^2+^-mediated ionic cross-linking between carboxylated agarose and tannic acid (TA). Each hydrogel component had an important role in its formation or biomedical application: the carboxylated agarose ensures good biocompatibility, while the tannic acid provides antibacterial and anti-inflammatory properties, which are essential for wound healing. Lastly, the Zn^2+^ ions contribute to the formation of the ionic cross-linking between the agarose chains and the tannic acid.

**Figure 4:**
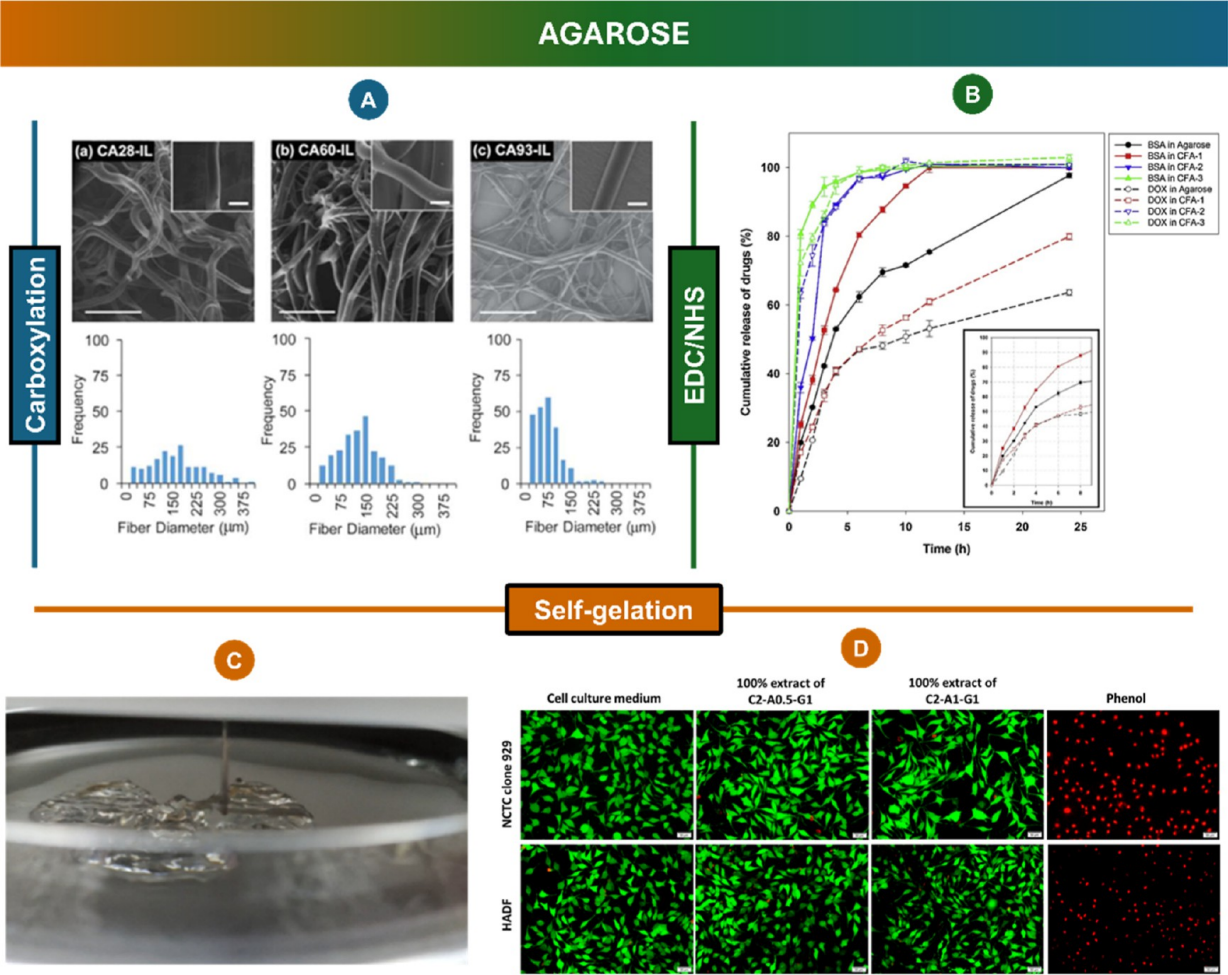
(A) Scanning electron micrographs and diameter determination of the electrospun fiber meshes. Reprinted with permission from.^157^ Copyright 2016 American Chemical Society; (B) drug release profiles for hydrogel-loaded BSA (filled symbols) and DOX (empty symbols) in PBS (pH 7.4) at 37 °C. Reprinted with permission from Elsevier;^164^ Bioprinting (C) and cytotoxicity (D) evaluation of the agarose-based bioink. Reproduced with permission from Elsevier.^158^

##### 3.2.2.2 EDC/NHS

The EDC/NHS carbodiimide coupling method enables the conjugation of biomolecules or other functional moieties to the agarose backbone. Specifically, it enables the insertion of dynamic motifs such as cyclodextrins, allowing the supramolecular interaction of agarose with other macromolecules. Recently, a cyclodextrin (β-CD)-functionalized agarose derivative was synthesized by EDC/NHS coupling reaction to obtain a hydrogel with lower stiffness than agarose gel and low gelling temperature, holding great promise to be used as a drug carrier.^164^ In fact, it was shown that the hydrogel could be used both for the complete release of BSA, which cannot complex with β-CD, and for the sustained release of doxorubicin (DOX), through inclusion complexation with β-CD (Figure 4B).

##### 3.2.2.3 Agarose Self-Gelation

As highlighted above, agarose can undergo a sol–gel transition upon cooling. Having this in mind, efforts have been made to use this temperature-triggered transition in the development of hydrogels. In this regard, carboxymethyl cellulose, agarose, and gelatin have been combined to design a novel semi-interpenetrated network-based thermoresponsive biomaterial ink for 3D printing^158^ (Figure 4C). Morphological studies revealed that an increase in the concentration of gelatin resulted in an increased pore size. A similar trend was observed when the agarose concentration was increased. A temperature-dependent rheology test revealed that the sol–gel transition occurred between 36 and 26 °C. Lastly, *in vitro* assays demonstrated that the biomaterial inks had more than 70% of viable cells, being highly promising for cell culture and tissue engineering applications (Figure 4D). In addition to its role in thermoresponsive biomaterial inks, agarose can also be used to fabricate a microparticle support bath for 3D printing.^165^ The fabrication of agarose microparticles is achieved by coiling a boiling solution of agarose containing calcium chloride, yielding particles with diameters ranging from 2 to 11 μm. The microparticle support bath exhibits good mechanical and rheological properties as well as good robustness to suspend gravity-defying structures.^165,166^ In a different approach, agarose films containing silver nanoparticles were produced, targeting the development of novel and economically competitive biomaterials denoting antimicrobial activity.^167^ The presence of calcium ascorbate, used in the production of silver nanoparticles, proved to have an important role in the overall features of the agarose films, such as thickness and tensile strength, and even in their antimicrobial activity against *S. aureus*.

### 3.3 Laminarin

#### 3.3.1 Chemical Modifications

Despite the lack of reactive functional groups in the LAM backbone, synthetic modifications can facilitate the introduction of structural arrangements that confer bioactive properties to this otherwise inert macromolecule. These tailored modifications can effectively transform LAM into a versatile platform for biomedical applications. LAM was modified with glycidyl methacrylate (Scheme 6A), which served as a chemical cross-linker for the preparation of hydrogels^168^ and microparticles.^169^ “Click” chemistry is another versatile procedure that includes the combination of activated molecules via a two-step coupling involving “click” functional groups (azide and alkynes), leading to the formation of a stable conjugate.^170^ The propargyl functionalization of LAM can be achieved through two distinct approaches: (i) reductive amination with propargylamine^171^ (Scheme 6B) or (ii) reaction with activated propargyl carbonylimidazole^172^ (Scheme 6C). In addition, 3-azidopropyl carbonylimidazole can also be used for the successful modification of LAM with azide functional moieties (Scheme 6C), which can then be coupled to terminal alkyne groups through CuAAC.^172^

**Scheme 6:**
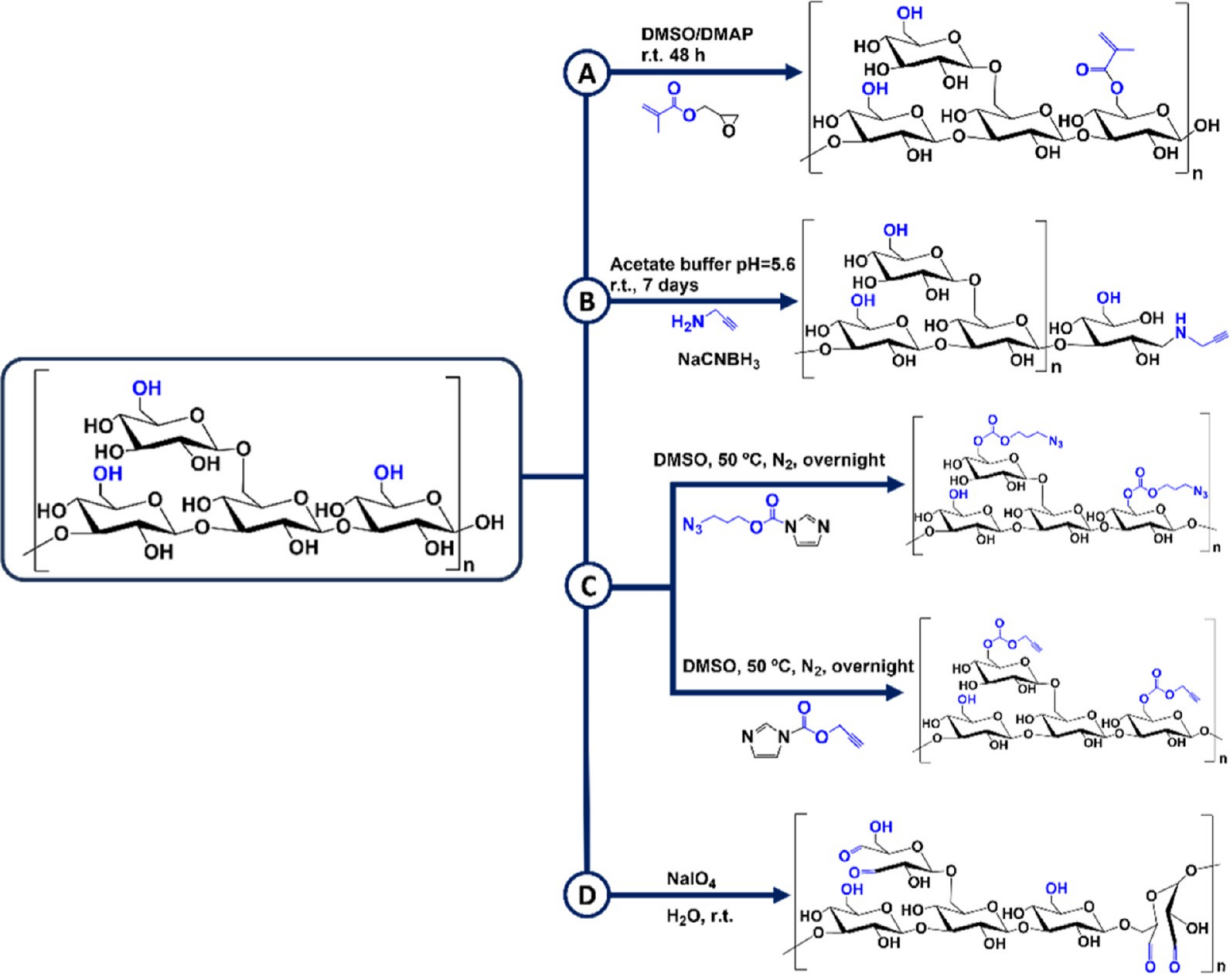
(A) LAM Methacrylation with Glycidyl Methacrylate; (B) Chemical Functionalization of LAM with a Propargyl Terminal by Reductive Amination; (C) Chemical Modification of LAM with a Propargyl and Azide Terminal Using Propargyl Carbonylimidazole and 3-Azidopropyl Carbonylimidazole, Respectively; and (D) LAM Oxidation with NaIO_4_

The structural configuration of LAM [β (1,3) and β­(1,6) intrachain linkage] is amenable to periodate-mediated oxidation, which is known to occur between the vicinal diols oriented in equatorial–equatorial or axial–equatorial positions.^173^ In this regard, the oxidation of LAM has been attempted by tuning the stoichiometric ratio of sodium periodate (NaIO_4_) in relation to the OH groups (Scheme 6D).^174^ The conversion of vicinal diols to dialdehyde moieties in LAM monomers could be precisely controlled to obtain different degrees of oxidation degrees.

Recently, the chemical functionalization of LAM with specific chemical moieties, such as allyl, amine, carboxylic acid, thiol, aldehyde, or catechol groups, enabled the creation of a new library of modified LAM with potential biomedical applications due to distinct cross-linking mechanisms employed for the development of cytocompatible biomaterials.^175^ The introduction of an alkene terminal at the LAM backbone was achieved by nucleophilic substitution via reaction between the hydroxyl groups of LAM with an allylation agent and a strong base (Scheme 7A). The allyl terminal reacted with thiol-bearing compounds through thiol-Michael addition; thus distinct groups such as amine, carboxylic acid, and thiol terminals can be introduced in the LAM backbone. Due to their high reactivity, catechols and their oxidized form, i.e. quinone, have attracted considerable attention toward the creation of novel biomaterials. Having this in mind, amine and carboxylic acid LAM derivatives were coupled with 3,4-dihydroxybenzoic acid or dopamine hydrochloride using the well-established EDC/NHS carbodiimide coupling chemistry.

**Scheme 7:**
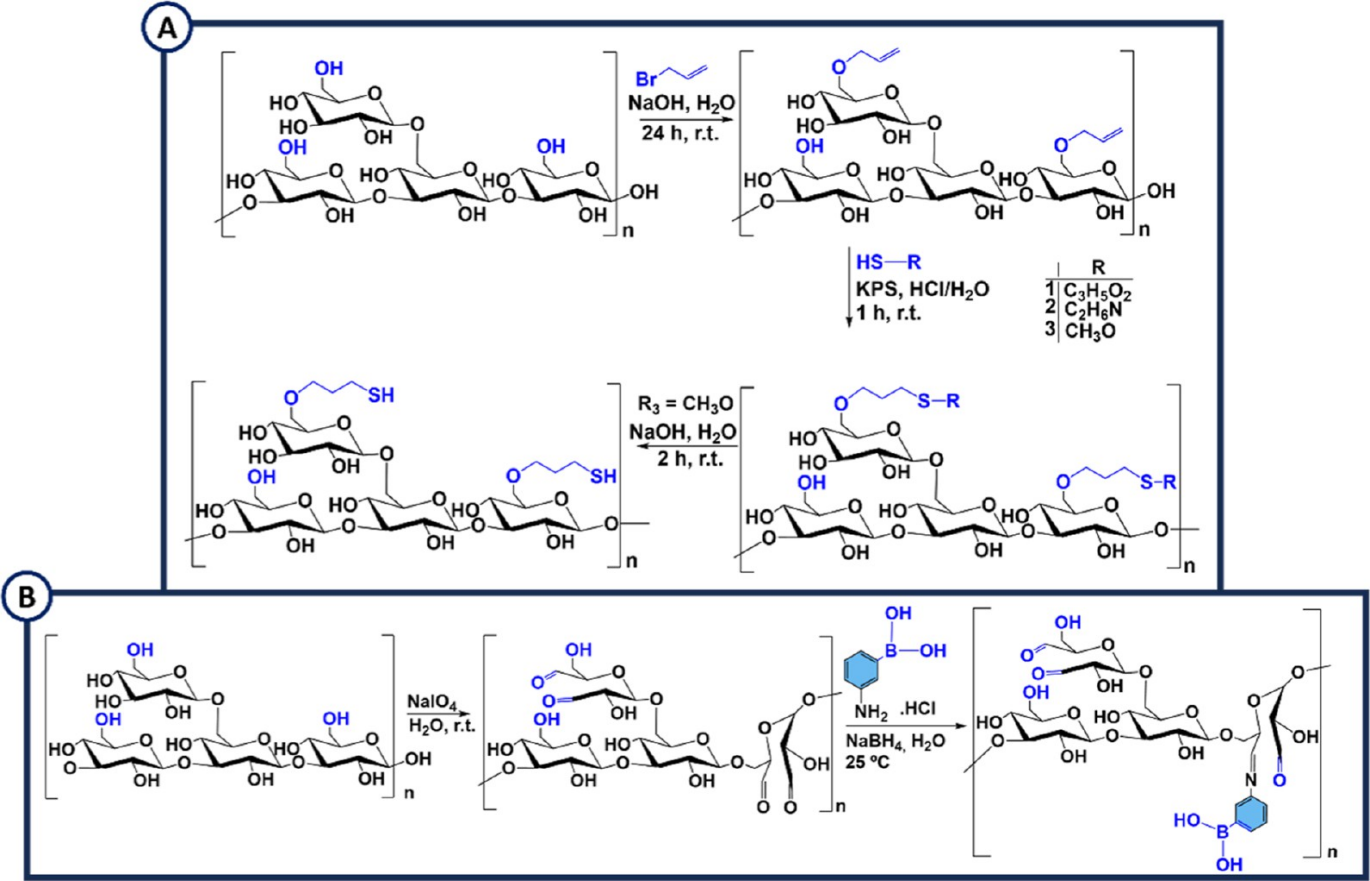
(A) Chemical Modification of LAM with an Allyl, Carboxylic Acid (R_1_), Amine (R_2_) and Thiol Terminal (R_3_); (B) Synthesis of LAM Functionalized with 3-Aminophenylboronic Acid Moieties

The chemical modification of LAM with metals by its complexation with strontium^176^ and by oxa-Michael addition reaction using divinyl sulfone as a cross-linker^177^ was recently proposed for the fabrication of LAM-based metal complexes, microgels, and nanogels. In addition, to impart dynamic properties to LAM, its modification with boronic acid groups was performed via a two-step reaction.^178^ Initially, LAM was oxidized at the C-3 and C-4 *cis*-diols with NaIO_4_, followed by the reaction of the oxidized product with phenylboronic acid, allowing the formation of a phenylboronic acid-modified LAM derivative (Scheme 7B). The boronic acid moieties enabled cross-linking with diols by dynamic, pH-responsive, and cyclic boronate ester bonds, resulting in injectable, self-healing, stimuli-responsive,^178^ and printable biomaterials.^179^

#### 3.3.2 Biomedical Applications

##### 3.3.2.1 Methacrylation

The modulation of the physical and biomedical properties of LAM can be achieved by its chemical functionalization with reactive and functional groups such as methacrylic,^169,175,180^ allyl, amine, carboxylic acid, thiol, aldehyde, catechol,^175^ azide, and/or alkyne terminal groups.^172^ The chemical functionalization of LAM with methacrylic groups enables the fabrication of hydrogels that can be successfully used for cell encapsulation and drug delivery. In this regard, the formation of an antimicrobial and high strength polymer hydrogel, prepared by the radical copolymerization of methacrylated LAM, *N*-acryloyl glycinamide (NAGA), and 1-vinyl-1,2,4-triazole (VTZ), was reported as having the ability to support cell adhesion and proliferation.^181^ The synergistic effect between the physical cross-linking, ensured by the hydrogen bonding interactions of the dual amide residues of NAGA, and the chemical cross-linking imparted by the methacrylated LAM, enabled the development of hybrid hydrogels with outstanding mechanical properties, achieving up to 3.2 MPa of compressive strength while maintaining high stretchability (650%). Additionally, the morphology of the hydrogel undergoes dynamic temporal changes upon hydration. Initially, the hydrogel only retains a small number of water molecules. After a determinate period of time, more water molecules start to enter the hydrogel network, culminating in a macroscopic enlargement of its diameter. Moreover, when immersed in a cellulase solution, the methacrylated LAM part of the hydrogel undergoes enzymatic degradation, while the supramolecular interactions preserve the hydrogel’s structural integrity, thereby increasing its porosity. The methacrylation of LAM was also used to develop microparticles for cell adhesion and expansion via microfluidics coupled to a source of UV light.^169^ In order to achieve this purpose, the microparticles were loaded with human platelet lysates (PL) and then coupled to an adhesive RGD peptide (Arg-Gly-Asp). The coupled peptide moieties dictated cell adhesiveness, while the PL was responsible for cell expansion (Figure 5A).

**Figure 5:**
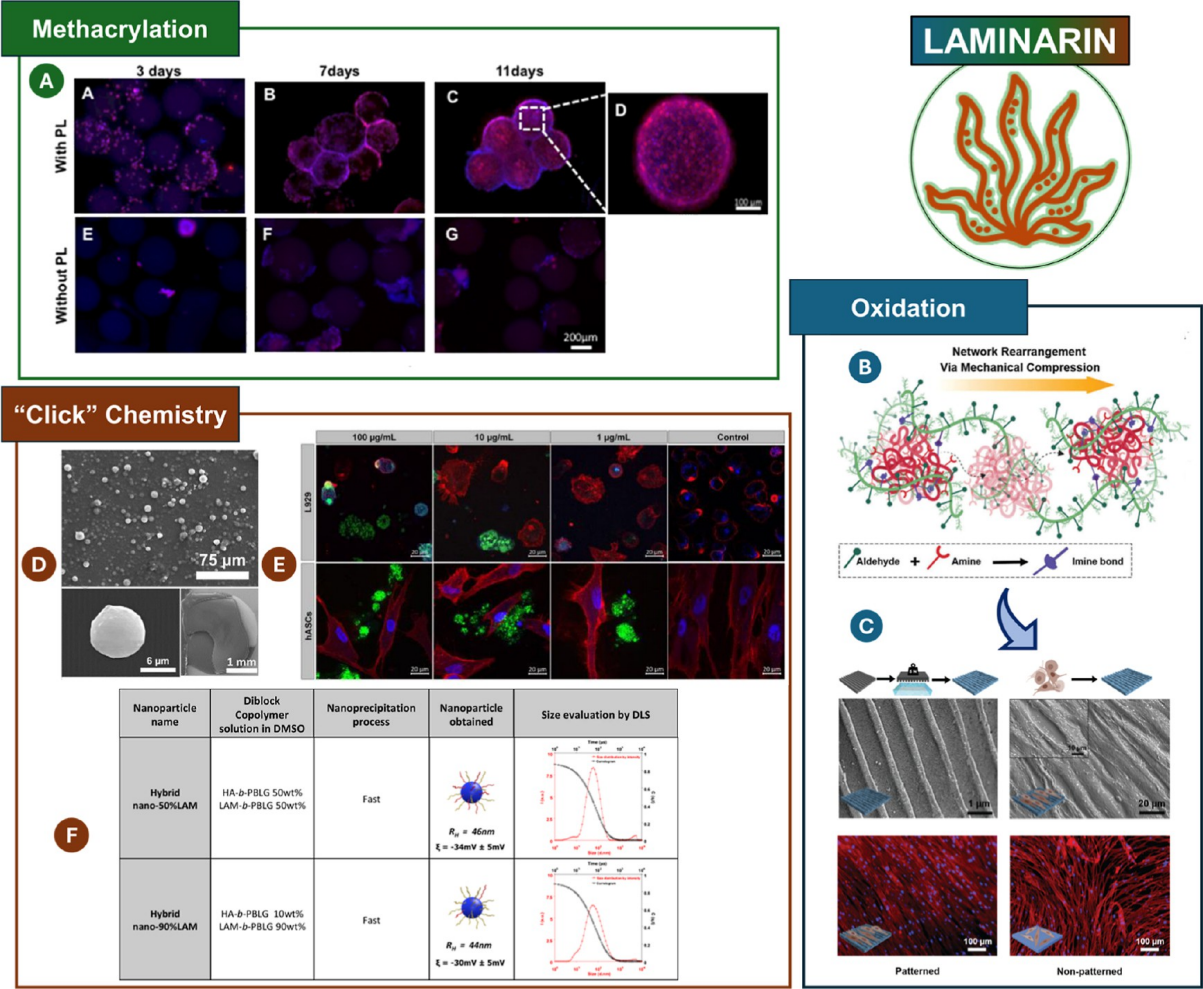
(A) DAPI/Phalloidin staining of L929 cells seeded on top of the methacrylated LAM microparticles with and without encapsulated PL. Reprinted with permission from Elsevier.^169^ (B) Dynamic covalently adaptable hydrogels formed through Schiff base reactions; (C) SEM imaging of the nanopatterned hydrogels before and after the attachment of hASCs (top). Fluorescence analysis of hASCs attached to nanopatterned and nonpatterned hydrogels; the F-actin is stained in red and the cell nucleus stained in blue (bottom). Reprinted with permission from.[Bibr ref174] 2020 WILEY-VCH Verlag GmbH & Co. KGaA, Weinheim; (D) SEM analysis of the modified and unmodified LAM microparticles; (E) fluorescence images of L929 fibroblasts and hASCs after 1 day of incubation with different microparticle concentrations. LAM microparticles were stained in green, the cell membrane stained in red, and the cell nucleus stained in blue. Reprinted with permission from Oxford University Press;^172^ (F) hydrodynamic radius (RH) and zeta potential ξ of copolymer-based nanoparticles obtained by nanoprecipitation. Reprinted with permission from.^171^ Copyright 2018 Nature.

##### 3.3.2.2 Oxidation

Although not naturally present in the LAM backbone, aldehydes generated through the oxidation of LAM can form Schiff base reactions when conjugated with amines. Lavrador et al.^174^ developed an amine-reactive oxidized-LAM that was further cross-linked with gelatin, resulting in the formation of extracellular matrix (ECM)-mimetic hydrogels for programmable cell organization (Figure 5B,C). Moreover, by varying the aldehyde-to-amine ratio, it was possible to control the cross-linking kinetics, viscoelastic properties, and degradation profile of the hydrogels. Additionally, the mechanochemical features of the cross-linked hydrogels offer a straightforward approach to creating a micro- or nanotopography that mimics the ECM microenvironment, thereby improving cell adhesion, proliferation, and organization. On the other hand, the functionalization of oxidized-LAM with boronic acid moieties enabled the development of LAM-based hydrogels that exhibited tunable mechanical properties with shear-thinning and rapid self-healing characteristics, responsiveness to ROS, and cytocompatibility.^114^

##### 3.3.2.3 “Click” Chemistry

LAM can be successfully modified with either azide or alkyne terminal groups to further undergo “click” chemistry reaction with any other macromolecule denoting alkyne or azide moieties, respectively. The “click” reaction between alkyne- and azide-modified LAM enabled the development of biodegradable microparticles with tunable size (Figure 5D,E) that could be used as drug delivery systems.^172^ In fact, the microparticles were able to release 40% of fluorescein isothiocyanate labeled-dextran after 24 h and experienced full degradability after 11 days under physiological conditions. In addition, the microparticles were revealed to be biocompatible toward human adipose stem cells (hASCs) and mouse fibroblast cells (L929). Using the same chemical reaction, LAM and HA were successfully modified with hydrophobic and helical poly­(γ-benzyl-l-glutamate) (PBLG) chains to construct self-assembled nanostructures using a nanoprecipitation process (Figure 5F).^171^ The nanoparticles assembled by the nanoprecipitation of both LAM and HA copolymers in different ratios denoted hydrodynamic radius ranging from 44 to 46 nm. Additionally, protein interaction assays (with CD44 and Dectin-1) revealed that the nanoparticle–protein interaction can be tuned by the particle structure and composition. This study demonstrated that it is possible to design multifunctional nanoparticles with tunable biological properties by resorting to a simple and straightforward approach such as co-nanoprecipitation.

### 3.4 Hyaluronic Acid

#### 3.4.1 Chemical Modifications

The HA biopolymer is a nonsulfated GAG ubiquitous in the ECM of tissues and organs, playing an important role in cell signal transduction and wound healing.^182^ The carboxylic acid, the primary and secondary hydroxyl groups, and the *N*-acetyl group (following deamidation) have all been widely studied regarding the chemical modifications of HA.^183^ The –COOH groups have been modified by carbodiimide-mediated reactions,^184,185^ esterification,^186^ and amidation;^187^ the –OH groups have been modified by etherification, divinylsulfone cross-linking, esterification, and bis-epoxide cross-linking.^183^ Furthermore, the diol groups can be easily oxidized to yield reactive aldehyde groups, which could then be used to make amide bonds through Schiff base reactions.^188^

The methacrylation reaction is one of the most widely employed chemical reactions for converting polymers into biomaterials. Nonetheless, introducing the C=C bond into the polymer backbone enables other types of chemical reactions, as well. In particular, methacrylated HA-based hydrogels (MeHA) were cross-linked using two different methods: a redox system (APS/TEMED) and Michael addition between the methacrylic groups of HA and the thiol groups of a metalloproteinase 7 (MMP7)-degradable peptide.^189^ The redox cross-linking system produced hydrogels with higher stiffness, which exhibited less swelling and a slower degradation rate. Furthermore, as the concentration of the MMP7-degradable peptide increased, the gelation time decreased, with the same trend observed for the degradation rate. Lastly, the functionalization of methacrylated HA with a CS-binding peptide was developed using a thiol-methacrylate reaction (Scheme 8). Unlike the previous examples, this functionalization resulted in increased gelation kinetics as well as a decreased hydrogel cross-linking density.

**Scheme 8:**
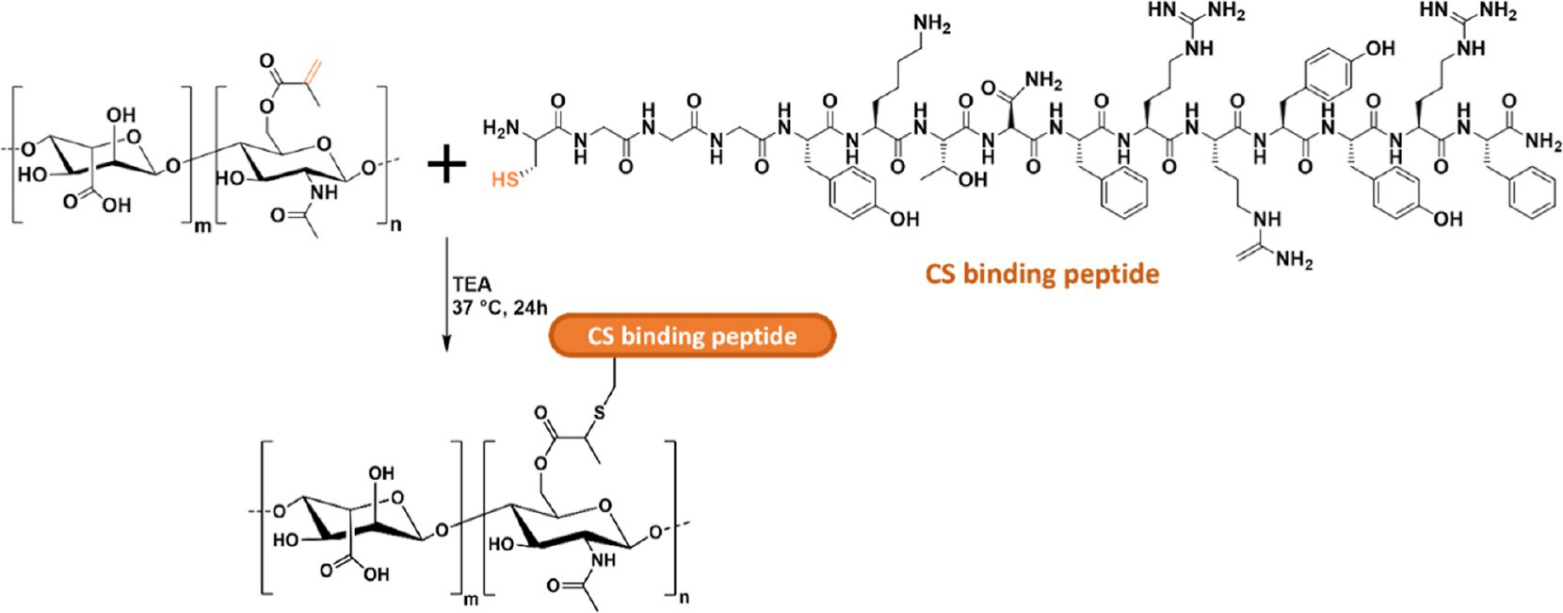
Modification of Methacrylated HA (MeHA) with the CS Binding Peptide Achieved by Thiol-Methacrylate Chemical Reaction

In a different approach, the modification of the carboxylic acid group of HA with furylmethylamine via amidation by resorting to 4-(4,6-dimethoxy-1,3,5-triazin-2-yl)-4-methyl-morpholinium chloride (DMTMM) as coupling agent was also reported^190^ (Scheme 9). This HA-derivative was chemically modified with adipicdihydrazide by amidation flowing the same protocol. Lastly, oxidized HA-Furan was synthesized by the oxidation of HA with NaIO_4_. The developed hydrogel was formed by two different reactions; first, the aldehydes established acyl hydrazone bonds with the amine groups of the adipicdihydrazide, and the second reaction was formed by reversible Diels–alder “click” chemistry between the furan groups of HA and the maleimide groups in the maleimide-PEG-maleimide (mal-PEG-mal) (Scheme 9). Other coupling agents can be used for the same purpose. Very recently, HA was functionalized with dibenzocyclooctyne-poly­(ethylene glycol)­4-amine (BCO-PEG4-NH2) using (benzotriazol-1-yloxy)­tris­(dimethylamino)­phosphonium hexafluorophosphate (BOP), a well-known peptide coupling reagent.^191^ The modified HA-DBCO was then assembled with metabolically engineered cell–surface glycoproteins bearing azide groups via copper-free strain-promoted [3 + 2] azide–alkyne cycloaddition (SPAAC). This approach enables the creation of living materials where the natural polymers make up only a small fraction of the total components, in contrast with traditional biomaterials, where the polymer fraction constitutes the majority.

**Scheme 9:**
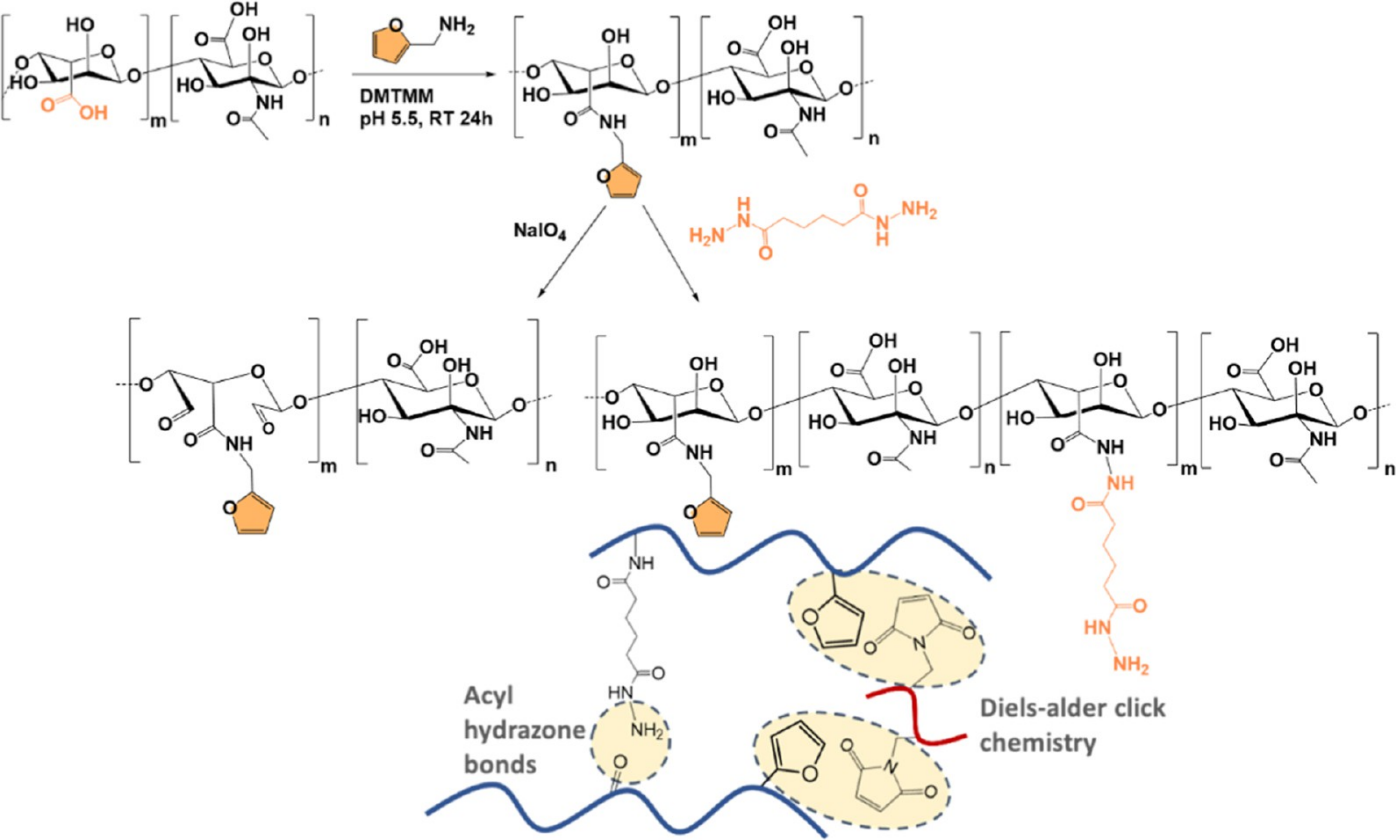
Chemical Routes Used to Modify HA Include Furan Functionalization, Adipicdihydrazide Conjugation, and HA Oxidation^a^

a Chemical bonds established during hydrogel formation between modified HA and maleimide-PEG-maleimide (mal-PEG-mal): the HA chains chemical modified with furylmethylamine and adipicdihydrazide are presented in blue and the chains of mal-PEG-mal are illustrated in red.

The introduction of dynamic and reversible noncovalent bonding has been gathering considerable attention and allows a polymeric network to adapt itself in response to external stimuli.^192^ In this regard, a self-assembled HA hydrogel was developed by exploring host–guest interactions between adamantane-modified HA and β-cyclodextrin-modified HA.^193^ The authors started by functionalizing HA with 1-adamantane acetic acid by esterification using DMAP as a nucleophilic base catalyst. The synthesized HA-adamantane worked as a guest macromer. Next, 6-(6-aminohexyl)­amino-6-deoxy-β-cyclodextrin (host) was synthesized for further conjugation with HA through amidation (Scheme 10).

**Scheme 10:**
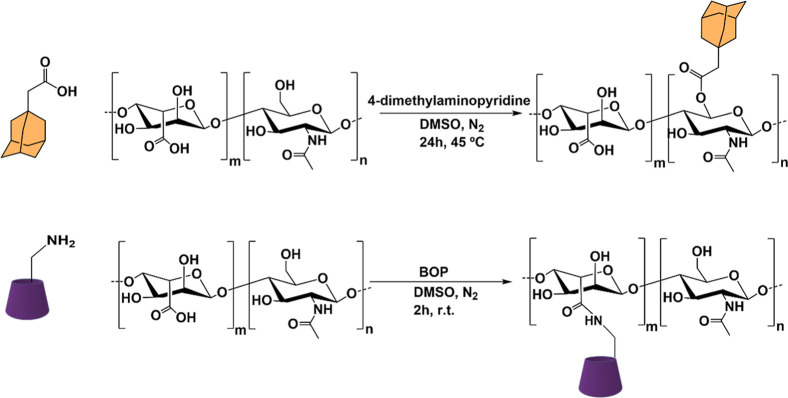
Chemical Modification of HA with Both Adamantane and Cyclodextrin to Develop a Supramolecular Self-Assembling Host–Guest Hydrogel

Recently, our group reported the molecular design, synthesis, and development of a dynamic multicomponent G-quadruplex system encompassing low MW HA, guanosine, and potassium chloride.^194^ The HA was functionalized with 3-aminophenylboronic acid units (HA-PBA) using DMTMM as a coupling agent. Then, the G-quartet monomers, formed through supramolecular Hoogsteen-type hydrogen bonding interactions between four guanosine units, were coupled to HA-PBA via dynamic and reversible cyclic boronate ester bonds. Despite the dynamic properties of G-quadruplex hydrogels, which make them highly appealing for biomedical applications, guanosine is poorly soluble in water. Therefore, hydrogel formation requires heating to dissolve guanosine. This heating process poses a significant challenge for cell encapsulation, as elevated temperatures can severely affect cell viability and impair their biological functions. To address this issue, other guanosine derivatives, such as guanosine monophosphate, have been used as powerful alternatives due to their higher solubility in aqueous environments, thereby overcoming the need for heating during the gelation process.

#### 3.4.2 Biomedical Applications

##### 3.4.2.1 Carbodiimide-Mediated Reactions

HA has a key role in a variety of biological processes, including cell proliferation and differentiation and helps to maintain the ECM’s physiological balance by providing hydration, lubrification, and matrix homeostasis.^195^ As such, HA is one of the most used marine polysaccharides in the biomaterial field. As aforementioned, HA can be chemically modified to impart it with a variety of physical properties, including improved mechanical features.^196^ Table 1 describes the most common chemical modifications applied to HA toward engineering novel biomaterials. For instance, a breast cancer ECM-mimetic HA hydrogel, obtained from two HA derivatives, one that was oxidized and another one encompassing methacrylic moieties and hydrazone molecules that were coupled to HA through EDC/NHS coupling chemistry, was proposed to support the growth and clustering of breast cancer MCF-7 cells.^197^ In addition, the vascular endothelial growth factor (VEGF), interleukin 8 (IL-8), and basic fibroblast growth factor (bFGF) expression levels were considerably higher in the 3D hydrogel-cultured cells than those in 2D culture platforms. The same chemistry was employed to modify HA with dopamine molecules aiming to develop an adhesive electrostatic-driven multilayered membrane by combining oppositely charged CHT, ALG and dopamine-grafted HA.^198^ The membranes containing dopamine-grafted HA presented a more porous morphology than the dopamine-free ones as well as a higher average thickness, greater water uptake and improved mechanical strength and cell adhesion. In addition, *in vivo* assays revealed that the dopamine-containing membranes presented the lowest inflammation level in the injured rat when compared with the dopamine-free membranes. Dynamic HA-functionalized G-quadruplex hydrogels denoting injectable, thermo-reversible, conductive, and self-healing properties were also assembled by dynamic boronate ester bonds in which the boronic acid moieties were coupled to HA via a DMTMM coupling reaction^194^ (Figure 6A). The intrinsic instability of the G-quadruplex structures was used to bioengineer size- and shape-tunable perfusable microchannels after being embedded in a gelatin methacrylated photo-cross-linkable supporting 3D matrix (Figure 6B). The microchannel-embedded 3D constructs denoted enhanced cell viability when compared to the bulk hydrogels and could be translated to virtually any kind of photo-cross-linkable supporting matrix, holding great promise for being used in drug delivery and modular TERM strategies.

**Figure 6:**
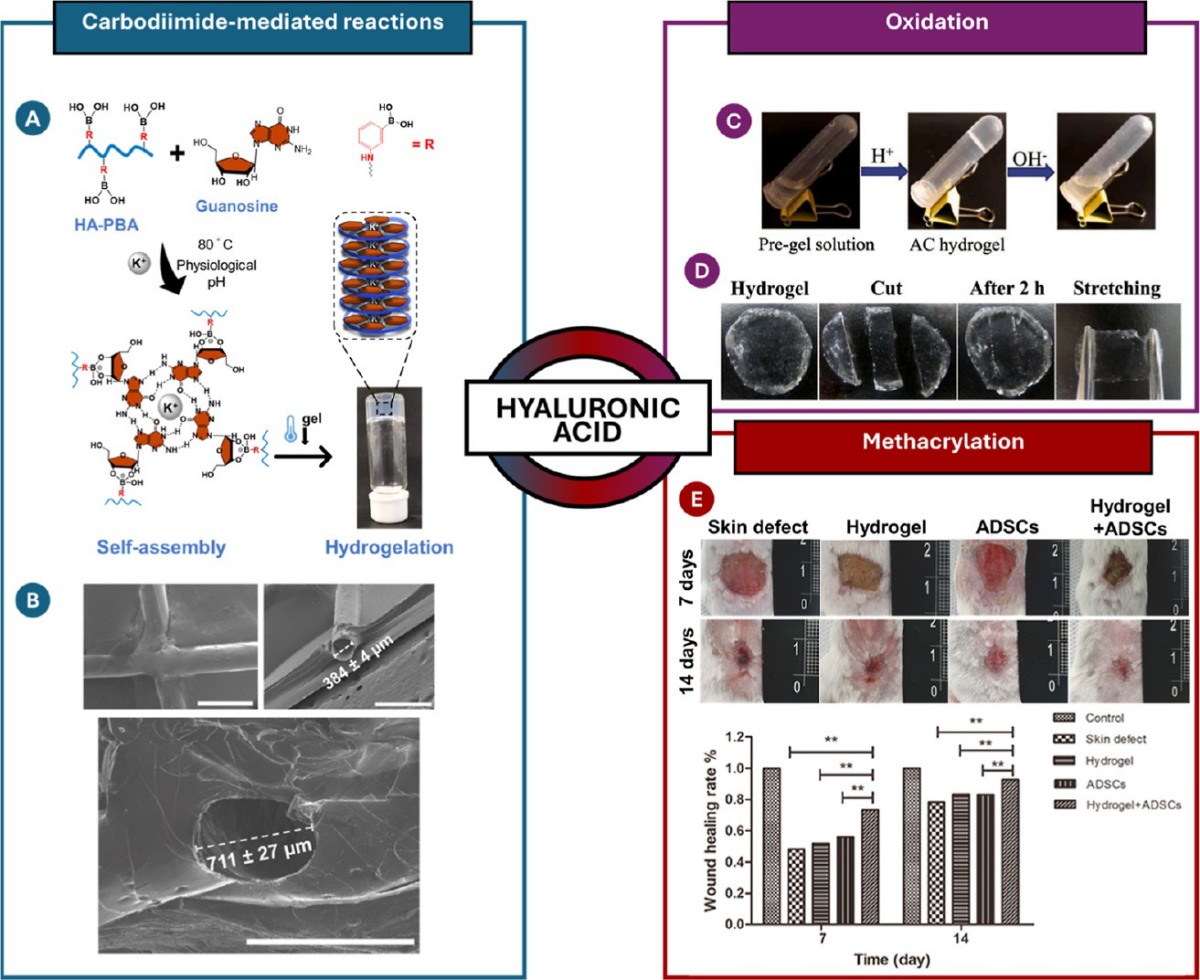
(A) Schematic illustration of the G-quadruplex hydrogel formation; (B) SEM images of the interconnected hollow channels within a methacrylated gelatin matrix. Reprinted with permission from.^194^ Copyright 2023 American Chemical Society; pH responsiveness (C) and self-healing ability (D) of the Cys-functionalized HA hydrogels. Reprinted with permission from Elsevier;^199^ (E) the potential of dopamine-methacrylated HA hydrogels for wound healing. Reproduced with permission from.^201^ Copyright 2022 Nature.

##### 3.4.2.2 Oxidation

Although not naturally present in the HA backbone, aldehydes generated by oxidation allow the conjugation of other molecules to HA or its cross-linking with other biomacromolecules. For example, this reaction has been used to allow the cross-linking of oxidized-HA with the amine groups of a modified HA derivative, yielding a novel composite hydrogel encompassing pH-sensitive nanoparticles loaded with VEGF.^190^ It was found that higher polymer concentration (10% (w/v)) or alkaline pH (pH 8) led to a reduction in the gelation time and to a decrease in the swelling ratio. Following the dispersion of VEGF-loaded nanoparticles into the oxidized-HA fraction, the composite hydrogel demonstrated the ability to sustain cell activity and promote cell growth. Another example pertains to the development of HA hydrogels denoting improved mechanical properties by cross-linking oxidized HA with cystamine dihydrochloride (Cys) via the Schiff base reaction.^199^ Due to the presence of dynamic imine bonds, the hydrogel was sensitive to the pH environment (Figure 6C) and showed excellent self-healing ability (Figure 6D). Oxidized HA can also be directly cross-linked with other biomacromolecules (e.g., CHT biopolymer) through the Schiff base reaction.^200^ It was found that the gelation time decreased while increasing the oxidation degree; in addition, increasing ratios of CHT resulted in hydrogels with thicker pore walls, enabling a slower degradation rate.

##### 3.4.2.3 Methacrylation

The incorporation of methacrylic moieties onto a polysaccharide’s backbone not only allows its easy transformation into a biomaterial but also facilitates its functionalization with other molecules via thiol-methacrylate chemical reaction. For instance, methacrylated HA cross-linked by a MMP7-degradable peptide was used to produce injectable hydrogels to repair cartilage injuries.^189^ It was found that by increasing the degree of modification of methacrylic HA, or its MW, the hydrogels showcased higher stiffness (higher elastic modulus, *G*′), lower swelling percentage, and a slower degradation profile. Although this trend is predictable and consistent with other polymers, a notable result was found when the cross-linking was conducted in a cell culture medium, resulting in an increase in the elastic modulus (*G*′ *ca*. 12 kPa), due to the electrostatic interactions established between the amine groups of MMP7-degradable peptide and the carboxylic acid present in the glutamine (present in the culture medium). Recently, methacrylated HA conjugated with dopamine revealed its potential for skin regeneration.^201^ The methacrylated HA hydrogel loaded with adipose-derived stem cells exhibited adhesion to skin wounds, facilitating cell differentiation. Furthermore, *in vivo* assays provided evidence that the cell-loaded hydrogel is more effective at promoting skin regeneration compared to cell-free hydrogels or *in situ* cell injection, as it enhances dermal collagen formation and neovascularization (Figure 6E).

### 3.5 Alginate

#### 3.5.1 Chemical Modifications

ALG is a biocompatible, safe, and nonimmunogenic biopolymer, which has been extensively studied in the biomaterials area due to its physicochemical and biological properties.^63^ ALG has been extensively used in TERM since it is a low-cost polymer, noncytotoxic, and an excellent precursor to 3D porous scaffolds. Recently, the functionalization of ALG with both methacrylic and cell adhesive moieties was demonstrated.^202^ Briefly, sodium counterions of ALG were substituted with tetrabutylammonium ions to facilitate the dissolution in organic solvents, namely, DMSO, followed by *in situ* conjugation of (i) cell adhesion molecules (CAM) through carbodiimide-mediated amide formation and (ii) methacrylate groups via a ring-opening nucleophilic reaction (Scheme 11). By combining these two chemical methodologies, it is possible to independently tune the cross-linking density and cell adhesive properties of the developed scaffold.

**Scheme 11:**
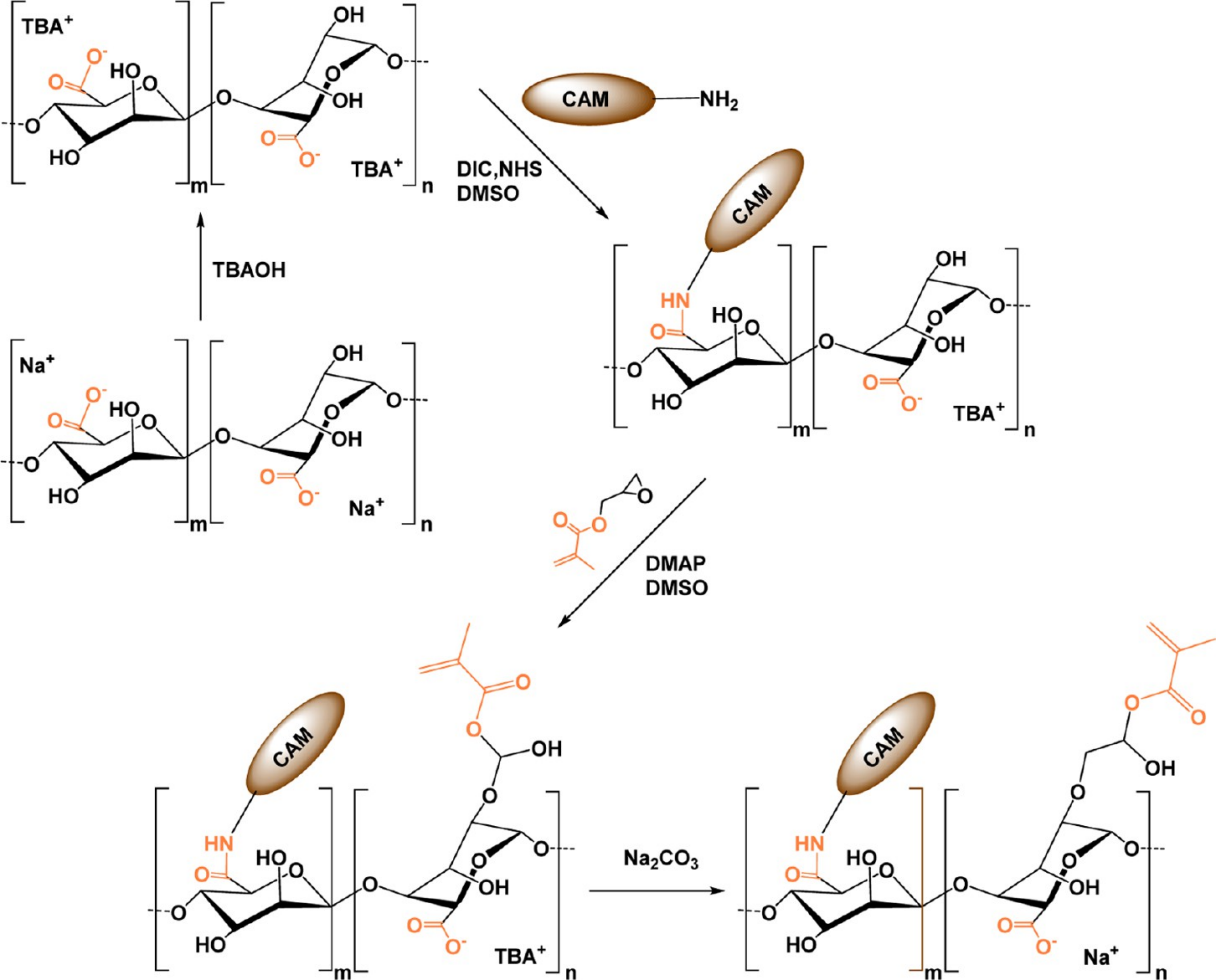
Synthesis of Methacrylated ALG Conjugated with a Cell Adhesion Molecule (CAM)

Instead of chemically modifying ALG with new functional and bioactive units, this polysaccharide can also benefit from its partial oxidation at the C-2 and C-3 positions to obtain reactive aldehyde groups, opening new avenues toward innovative biomaterials.^203^ The predominant method for achieving vicinal bond cleavage typically employs periodate salts, which induce the formation of aldehyde groups that could form Schiff base through conjugation with amine groups.^203^ Following the same Schiff base reaction, lotus-leaf-derived porous ALG membranes were developed by combining oxidized ALG with carboxymethyl CHT (Scheme 12, left panel). In a different approach, the methacrylation of ALG was accomplished by making use of 20-fold excess of methacrylic anhydride and its further oxidation resorting to NaIO_4_ ^204^ (Scheme 12, right panel). The insertion of the methacrylate groups enabled photo-cross-linking when mixed with an eosin Y-based photoinitiator and after exposure to visible green light, which offers a broader range of therapeutic possibilities with significantly lower risks compared to UV light. The generation of functional aldehyde groups allows the further establishment of imine bonds with proteins found in several tissues.

**Scheme 12:**
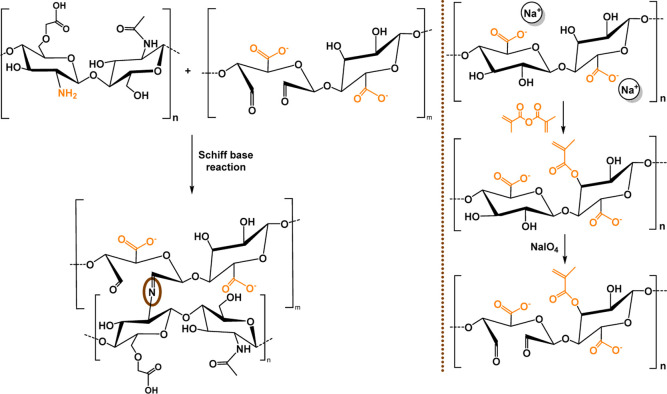
On the Left Is Illustrated the Approach Used to Construct Lotus Nanofibers-ALG Porous Membranes, by Shift Base Reaction between Oxidized ALG and Carboxymethyl CHT. On the Right Is Represented the Conjugation of Both Methacrylation and Oxidation of ALG to Develop a Tissue Sealant for Lung Repair

Sulfated polysaccharides have gained special attention in the biomedical field due to their ability to work as blood-compatible anticoagulants. The sulfation of ALG using chlorosulfonic acid was reported to achieve a substitution degree as high as 1.41; however, to avoid the side effects caused by the oversulfation, quaternary amine groups were attached by reacting ALG sulfates with 2,3-epoxypropyl trimethylammonium chloride. Since quaternary amine groups lower anticoagulant activity, the number of these groups must be controlled to avoid both oversulfation and significant decrease in the anticoagulant activity.^205^ By leveraging H_2_SO_4_ and *N*,*N*′-dicyclocarbodiimide (DCC) as sulfated agents, a novel heparin mimicking ALG sulfate derivatives (SASs) was synthesized.^206^ The novel derivative was also grafted with dopamine moieties (DA-*g*-SASs) by the EDC/NHS carbodiimide coupling chemistry reaction to impart adhesiveness to the material (Scheme 13A).

**Scheme 13:**
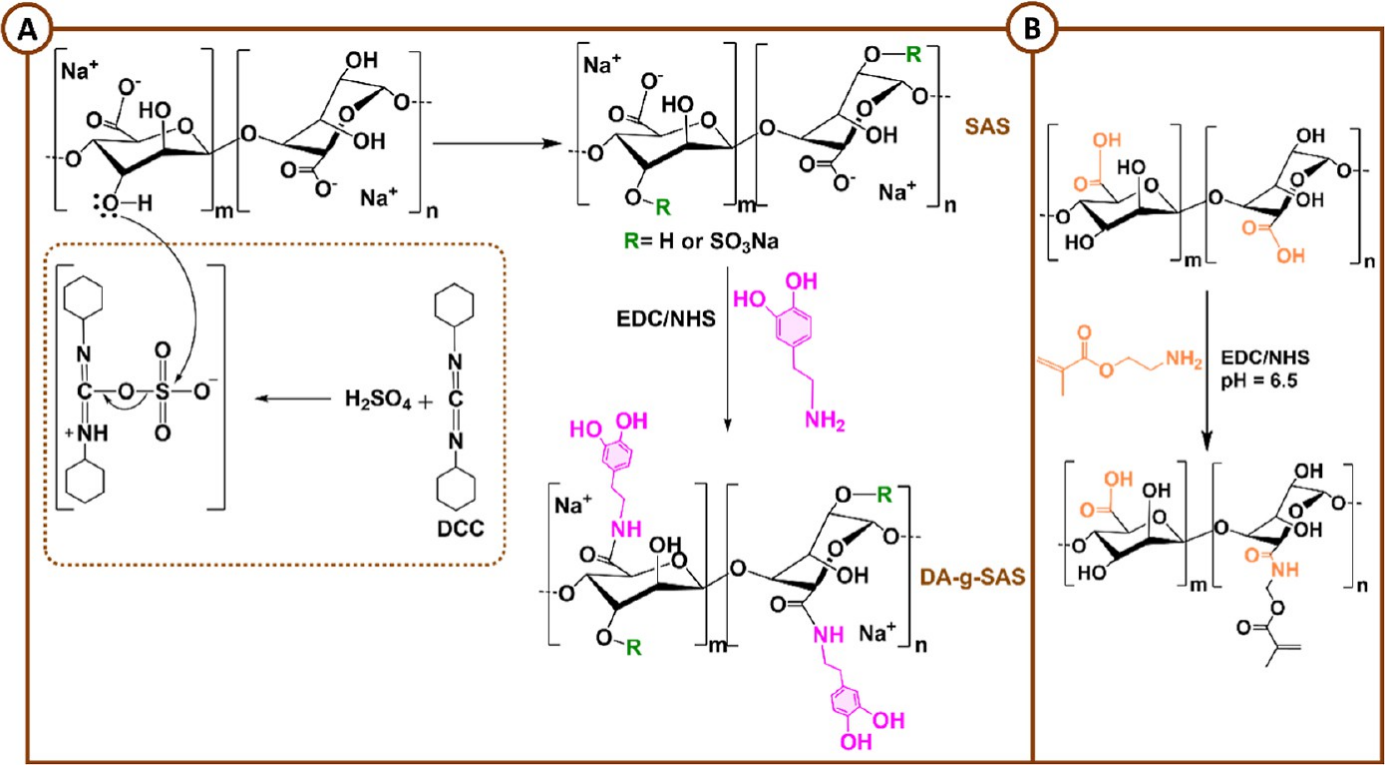
(A) Schematic Representation of the ALG Sulfates (SAS) and Dopamine-Grafted SASs (DA-g-SAS) Synthesis^a^ (B) Chemical Modification of ALG with 2-aminoethyl Methacrylate by an EDC/NHS Coupling Reaction

a The SO_3_Na groups (in green) were attached to the ALG backbone using DCC and H_2_SO_4_ as sulfated agents; in addition, by EDC/NHS coupling reaction the dopamine moieties (in pink) were grafted to the ALG sulfates (DA-g-SAS).

Unlike conventional hydrogels, cryogels allow better nutrient and oxygen diffusion due to their interconnected macropores. In addition, their densely cross-linked polymer walls impart them with exceptional elasticity and shape-memory properties.^207,208^ Having this in mind, ALG cryogels have been reported as biomaterial platforms to be delivered into the body through a conventional needle-shaped syringe injection.^208^ In opposition to hydrogels, which are commonly formed at room temperature through the presence of a photoinitiator and by exposure to UV-light, in this study, ALG was chemically modified with 2-aminoethyl methacrylate through EDC/NHS carbodiimide coupling chemistry to allow further radical polymerization by the addition of APS/TEMED initiators (Scheme 13B). Then, the mixture was frozen at −20 °C to generate an ice crystal template, which upon thawing gives rise to a macroporous network.

#### 3.5.2 Biomedical Applications

##### 3.5.2.1 EDC/NHS

Injectable biomaterials, mainly those to be implanted via minimally invasive procedures, are increasingly being pursued to minimize risks and complications associated with surgical implantation as well as patient pain and discomfort. Having this in mind, Mooney and co-workers developed an injectable ALG-based cryogel with excellent shape–memory properties.^208^ In fact, the developed cryogel could be shaped into diverse geometries and sizes and endure reversible deformations at strain levels exceeding 90%, achieving nearly complete geometric recovery upon injection through a small needle (16G) into the target site. In order to potentiate the biological response, cell assays were performed with ALG functionalized with a cell-adhesive peptide (RGD sequence), a common strategy when working with noncell adhesive matrices like ALG.^209^ With that in mind, *in vivo* assays revealed that these cryogels exhibited a sustained release of BSA over almost 3 months. Furthermore, the cryogels seeded with B16–F10 cells showcased improved mice’s survival rate and enhanced cell retention on the target site when compared to a standard cell injection procedure (Figure 7A). These injectable scaffolds have a lot of potential in biomedical applications, including as cell therapies or even as cancer vaccines.^210,211^ Despite the above-mentioned advantages of using cryogels as vehicles for advanced therapeutics, this type of scaffold does not enable cell encapsulation due to the cryogenic procedure needed to produce the ice template. On the other hand, and using the same type of reaction, a dual-functional ALG hydrogel containing both gelatin and RGD peptides was developed.^132^ The presence of these bioactive motifs in the ALG backbone enhanced cell adhesion and proliferation, opening new avenues for the design of cell culture 3D platforms.

**Figure 7:**
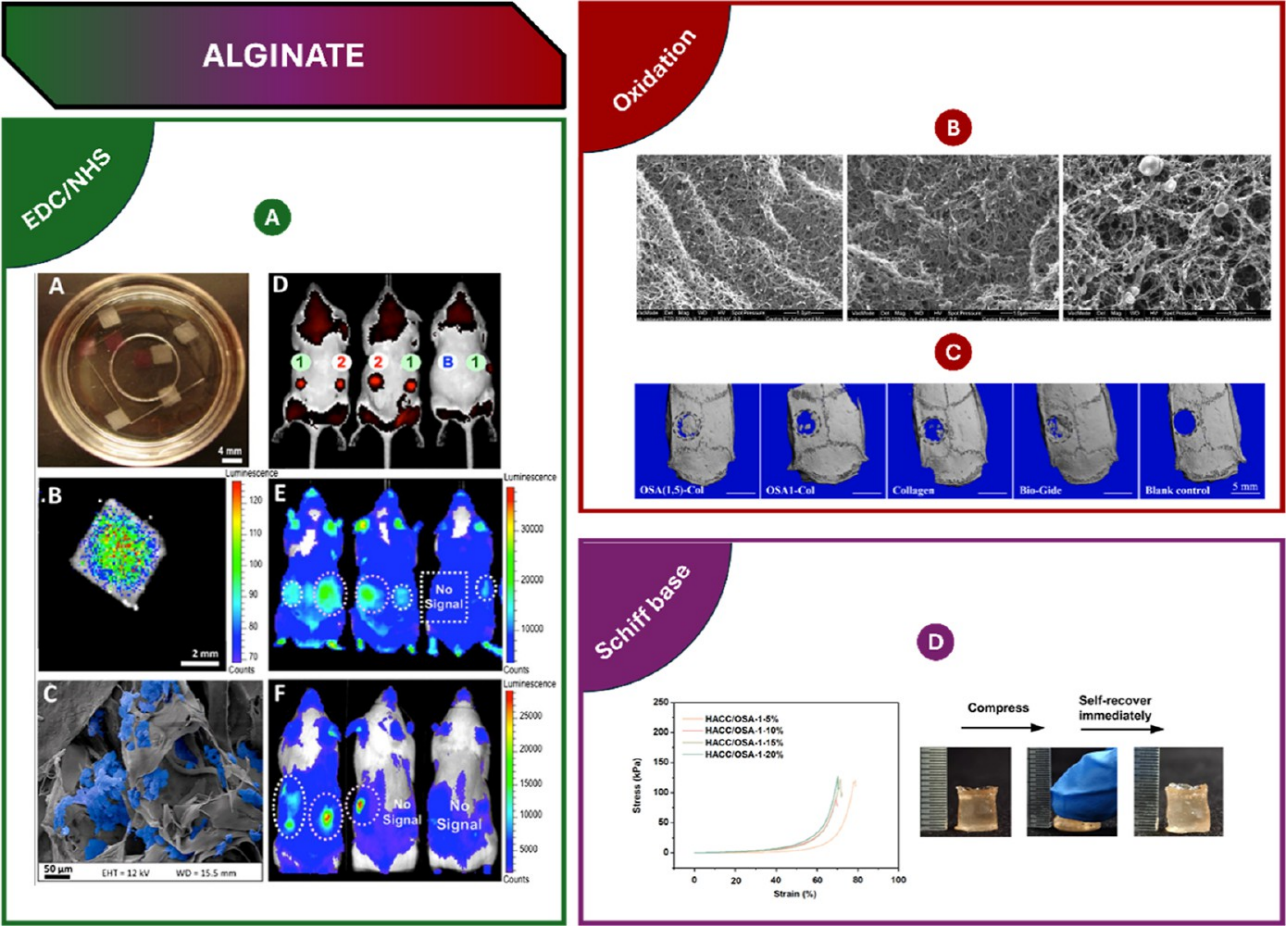
(A) The cryogels were loaded with cells and injected into the mice’s back to study *in situ* localization and cell retention. Reproduced with permission from.^208^ Copyright 2016 National Academy of Sciences; (B) SEM images showcasing the porosity of unmodified (left) and oxidized ALG hydrogels (middle, 2%; right, 5%). Reproduced with permission from.^203^ Copyright 2013, John Wiley and Sons; (C) microcomputed tomography (micro-CT) images of rat calvarial defects at 8 weeks postsurgery. Reproduced with permission from Elsevier;^213^ (D) stress–strain curves and recoverable compression test of the hydrogels based on CHT derivatives and oxidized ALG (HACC/OSA). Reprinted with permission from.^214^ Copyright 2023 American Chemical Society.

##### 3.5.2.2 Oxidation

As mentioned earlier, ALG is well-established as a biocompatible material for cell culture. However, it is imperative to modify native ALG to create scaffolds that closely emulate the physiological environment, thus allowing for optimal cell viability and function. In this regard, oxidized ALG-based hydrogels cross-linked with CaCl_2_ were developed as a biomimetic niche for limbal epithelial stem cells, being potentially applied in the treatment of corneal dysfunction.^203^ It was found that the oxidized ALG hydrogels exhibited larger internal pores when compared to unmodified ALG (Figure 7B), along with significantly lower stiffness, leading to increased cell viability. The same ALG-derivative has been also used to construct membranes.^212,213^ More specifically, a novel sponge-like bilayer membrane containing oxidized ALG and collagen (OSA-Col) was developed targeting bone tissue regeneration (Figure 7C). Mechanical tests revealed that the incorporation of oxidized ALG increased the compressive strength compared to its oxidized ALG-free counterpart. Additionally, the incorporation of this ALG derivative allowed control over the pore size, improved the swelling behavior, and slowed the degradability of collagen-based sponges. Lastly, the study revealed that the membranes with larger pore sizes showed a more pronounced osteogenic differentiation of bone marrow stromal cells.

##### 3.5.2.3 Schiff Bases

The appealing features of oxidized ALG turn it into a promising biopolymer for biomedical applications. Beyond the examples listed above, the oxidized ALG has also been used for conjugation with other polysaccharides via Schiff base reactions.^28,215,216^ In particular, a novel oxidized ALG hydrogel cross-linked with *N*-2-hydroxypropyl trimethylammonium chloride CHT via a Schiff base reaction was reported, revealing a short gelation time (74 s).^214^ The opposite charges of CHT and ALG can lead to complexation by electrostatic interactions. As such, to address this potential issue, the charges of both polysaccharides were shielded through the addition of NaCl. The mechanical tests revealed that the hydrogel had a compressive modulus of 22.5 kPa (Figure 7D). Furthermore, playing with the concentration of oxidized ALG (0.4–20%) did not result in a significant impact on the compressive ultimate stress of the material, although the compressive modulus was higher. In addition, the formulated hydrogel displayed self-healing behavior, excellent swelling, and antibacterial properties. Regarding this type of chemistry, the combination of oxidized ALG and gelatin is a common employed strategy to construct hydrogels,^28^ microcapsules,^217^ and 3D-printed hydrogels with suitable resolution (250 μm) for bioplotting aproaches.^218^ Upon optimization of the printing settings, a bioink encompassing MG-63 osteoblast-like cells was developed, revealing successful adhesion and proliferation of the encapsulated cells and angiogenic potential.^218^

### 3.6 Carrageenan

#### 3.6.1 Chemical Modifications

Despite the temperature-dependent gelation ability of κ-carrageenan, over time, the hydrogels undergo syneresis, a phenomenon by which the hydrogel spontaneously start to release water from the gel phase.^219^ This ability makes carrageenan widely used in the pharmaceutical industry;^220^ however, this process affects the rheological and mechanical properties,^221^ making the chemical manipulation of this polysaccharide in high demand. One of the first and most studied chemical routes to avoid carrageenan syneresis and enhance its gelation behavior is the cyclization reaction.^222^ In this reaction, the presence of a strong base (NaOH, 1M) at 80 °C catalyzes the cyclization of the α-d-galactose-6-sulfate units into 3,6-anhydro-α-d-galactose.^223^

More recently, graft copolymerization has also been proposed as a novel approach to improve the overall chemical properties of carrageenan, specifically to enhance mechanical stability and hydrophobicity and reduce syneresis. Synthetic polymers/groups like, dimethylaminoethyl methacrylate (DMAEMA),^224,225^ acrylic acid (AA),^224,225^ octyl chloride,^226^ among others^221^ have been grafted onto carrageenan. In fact, to aid the polymerization, the copolymerization of DMAEMA and AA onto κ-carrageenan through microwave irradiation using 4,4′-Azobis­(4-cyanovaleric acid) as a initiator was proposed.^224^ Later, the same authors took advantage of this novel carrageenan derivative to develop nanospheres for drug delivery^227^ by ionic cross-linking with iron ions. Using the same type of chemical route, carrageenan was grafted with synthetic polymers by free radical polymerization using ammonium persulfate as an initiator.^228^ Interestingly, by adding *N*–*Ń*-methylenebisacrylamide, the authors were able to achieve covalent cross-linking simultaneously with graft copolymerization, resulting in the hydrogel formation. The hydroxyl groups in the carrageenan backbone can be leveraged for esterification in the presence of an alcohol. Taking this into account, carrageenan was esterified with 1-chlorooctane in the presence of NaOH to enhance its hydrophobicity and ultimately improve its role as an emulsifier or stabilizer.^226^ Due to its structural similarities to heparin, a powerful blood anticoagulant, carboxymethyl carrageenan had gained significant interest among researchers.^229–231^ The chemical method used to obtain this derivative is consistent across different studies. Typically, carrageenan is dispersed in 2-propanol in the presence of NaOH, followed by the addition of monochloroacetic acid to initiate etherification. However, it is noteworthy that decreasing the reaction time from 4 ^231^ to 3 h^230^ yields higher degrees of substitution.

#### 3.6.2 Biomedical Applications

##### 3.6.2.1 Metal Coordination

Carrageenan is a very appealing polysaccharide for the design of biomaterials, mainly due to the excellent gel forming ability, especially in the presence of cations such as potassium (K^+^), sodium (Na^+^), and calcium (Ca^2+^);^232^ however, as described above, the resulting hydrogels still lack important features. To address this issue, zinc oxide (ZnO) and copper oxide (CuO) nanoparticles were incorporated into carrageenan and cross-linked with K^+^ ions to form a nanocomposite.^233^ The resulting nanocomposite hydrogels were revealed to have high swelling ratios; in particular, the incorporation of ZnO nanoparticles resulted in the highest increase as well as higher thermal stability. Regarding the mechanical properties, the presence of nanoparticles increases the hardness of the hydrogels; however, no significant increase was detected in stiffness. This increase was probably due to electrostatic interactions between the positive surface charge of the nanoparticles and the negative charge of carrageenan. In addition, the presence of the metallic nanoparticles also granted the biomaterial with notable antimicrobial activity against *Escherichia coli* and *Listeria monocytogenes*. Due to the appealing features of carrageenan, a variety of wound dressing materials have been developed;^26^ nonetheless, some properties can be modulated for better performance. For instance, films produced by solvent casting were designed resorting to κ-carrageenan, TA, and aluminum chloride.^234^ The resulting films were also loaded with simvastatin (an antihyperlipidemic drug with potent angiogenic properties) and Geranium oil (anti-inflammatory and antimicrobial). The results showcase that the films presented excellent mechanical strength, swelling rate, and appealing release kinetics. The synergistic effect between the loaded cargo granted the films antioxidant, anti-inflammatory, antibacterial, and angiogenic activities. Accordingly, *in vivo* tests reveal excellent epidermal regeneration with a wound contraction percentage of ca. 100% after 14 days.

##### 3.6.2.2 Graft Copolymerization

Given its thermoresponsive properties, carrageenan represents a promising approach for thermally triggered drug release. Based on this, triple-responsive magnetic nanoparticles with an iron oxide core (Fe_3_O_4_), coated with κ-carrageenan grafted with poly­(acrylic acid/dimethylaminoethyl methacrylate) (P­(AA/DMA)), were developed through microwave-assisted *in situ* precipitation and loaded with 5-Fluorouracil (5-FU) as an antineoplastic drug.^227^ The authors concluded that the release kinetics were more temperature- and pH-responsive; however, when stimulated by an alternating magnetic field, an accelerated release profile was detected, confirming the triple responsive ability of the nanostructures. However, the lower critical solution temperature detected was higher than the body’s physiological temperature, which can slow down the release profile when applied *in vivo*. Lastly, *in vitro* studies revealed that the nanospheres were biocompatible and the released cargo displayed high anticancer activity toward A549 cancer cells (lung cancer). With the same goal, Thakur and Singh^228^ developed a hydrogel through the graft copolymerization of carrageenan and poly­(vinylsulfonic acid) for the controlled release of vancomycin. Additionally, the authors incorporated polyacrylamide as a second cross-linking to form an interpenetrating polymer network. The resulting hydrogels exhibited nonthrombogenic and nonhemolytic properties. Furthermore, the material demonstrated mucoadhesive and antioxidant properties. Due to the vancomycin loading, the hydrogels also showed strong antibacterial activity against *P. aeruginosa*, *E. coli*, and *S. aureus*. Although the authors stated that the hydrogels did not exhibit cytotoxic effects, the results were not presented, making it difficult to draw definitive conclusions.

### 3.7 Fucoidan, Ulvan, Chondroitin Sulfate, and Dermatan Sulfate

#### 3.7.1 Chemical Modification

Acetylation involves the reaction between alcoholic hydroxyl groups and the carboxyl group of an acetylating agent and has been reported to significantly alter the properties of polysaccharides. More specifically, the acetylation of fucoidan not only enhances its antioxidant activity but also facilitates nanoparticle formation. By using acetic anhydride as the acetylating agent and formamide as a solvent, the fucoidan derivative was obtained, with approximately 87% of hydroxyl groups modified with hydrophobic acetyl moieties.^235^ In addition to acetic anhydride, phthalic acid anhydride and *N*-bromosuccinimide can also be used for the successful esterification of fucoidan.^236^ Beyond acetylation, the amination of fucoidan has also been reported as a pathway to enhance its anticoagulant properties. However, direct amination of fucoidan is not possible due to its heterogeneous nature. Instead, a preactivation step is needed, where a spacer molecule is introduced into the backbone of fucoidan by reacting it with epichlorohydrin. Subsequently, the product is aminated by dissolving it in a 30% ammonia solution.^237,238^

Unmodified fucoidan has been blended with ALG or other polymers to create physically cross-linked biomaterials, such as hydrogels^239,240^ or capsules.^241,242^ However, these systems suffer from a high rate of fucoidan diffusion due to the absence of covalent cross-linking. To address this limitation, Amin et al.^243^ recently reported the synthesis of methacrylated fucoidan using methacrylic anhydride. Since methacrylic anhydride reacts with water, generating acrylic acid as a byproduct, the authors used a 10% molar excess relative to the polysaccharide repeating unit. The reaction was carried out in a DMSO: water mixture (70:30 vol %), resulting in a degree of methacrylation of 12–13%.

Ulvan, DS, and CS have been also recognized as promising building blocks for designing ECM-mimetic advanced biomaterials due to their ability to interact with a variety of proteins and cell-expressing motifs.^34^ However, most of the time, they should be chemically modified to achieve more complex architectures with exquisite properties, enabling them to serve as advanced biomaterials. A good example of this is the methacrylation of ulvan; in order to enhance its methacrylation degree frequently, the reaction mixture is maintained at a slightly basic pH to neutralize the formed methacrylic acid, thus promoting the conjugation between ulvan and methacryloyl groups and reducing the extension of hydrolysis (Scheme 14A). The reaction temperature should also be controlled to limit the formation of side-products due to hydrolysis and thermally induced polymerization of the large excess of methacrylate groups present in the reaction mixture.^244^

**Scheme 14:**
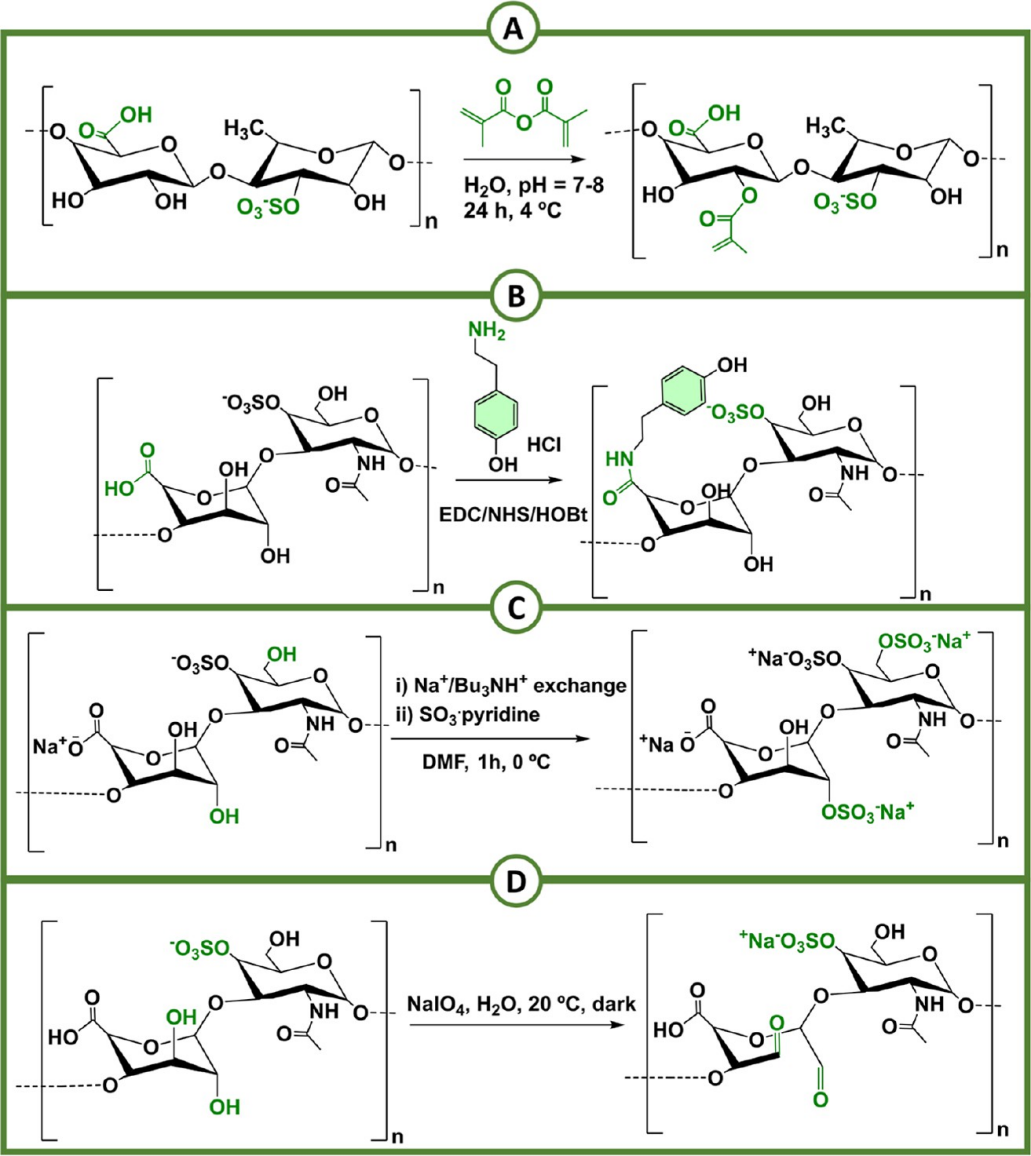
(A) Methacrylation of Ulvan; (B) Coupling Reaction of CS with Tyramine; (C) Regioselective O-Sulfation of CS with Sulfur Trioxide Complexes; (D) Oxidative Cleavage of CS

CS is another sulfated GAG with a chemical structure prone to be modified with a variety of molecules such as catechols, methacrylic units, or aldehydes.^245^ Furthermore, one of the most common functionalization of CS is achieved through the coupling between the GlcA carboxylic acid with amines or hydrazide moieties, resorting to carbodiimide reagents such as EDC, with the reaction usually being performed in slightly acidic conditions and in the presence of NHS or HOBt as protic additives (Scheme 14B).^246^ The coupling reaction of CS with different amines or hydrazides afforded functionalized polysaccharides for several biomedical applications,^247^ including drug delivery^248^ and tissue engineering.^249–251^

The CS and DS sulfation patterns could be modified using different synthetic routes; however, the per-*O*-sulfation should be avoided due to strong allergic-type response of these derivatives.^246^ Three distinct strategies are employed in the modification of CS: regioselective sulfation reactions, regioselective desulfation, and sulfation of appropriately protected polysaccharide derivatives. In fact, it was found that the sulfation regioselectivity followed a specific trend, GalNAc-O-6 ≫ GlcA-O-2 > GlcA-O-3 ≈ GalNAc-O-4 (Scheme 14C).^246^

As observed in many other polysaccharides, CS and DS can be easily oxidized, enabling additional conjugations with other (macro)­molecules. The oxidation of CS and DS can be split into three different subclasses: selective oxidation at the primary hydroxyl group of GalNAc, diketone formation from a vicinal diol, and oxidative cleavage of a vicinal diol.^246^ The oxidative cleavage of GlcA 2,3-diol to afford dialdehyde was achieved through the polymer treatment with different amounts of NaIO_4_ in water at 20 °C (Scheme 14D).^252,253^

In addition, the methacrylation of CS has also been explored as an attractive procedure to produce biomaterials.^254^ Generally, two different approaches can be employed: one utilizes methacrylic anhydride (MAA) while the other uses glycidyl methacrylate (GMA), both usually under alkaline conditions. However, when resorting to MAA, due to its low efficiency and propensity to hydrolyze, a large molar excess is needed. Alternatively, the fast transesterification of GMA also allowed the formation of methacrylate CS as main product (Scheme 15).^255,256^ Since both GMA and MMA have poor water miscibility, regardless of the reagent employed, the aqueous environment leads to low reproducibility.^257^ Alternatively, DMSO can be used to produce homogeneous-phase media for the reaction to occur; however, due to the low solubility of CS in organic solvents, this procedure requires an ion-exchange process in which the sodium counterion of CS is replaced by TBA^+^ cations.^258^ More recently, it was found that in DMSO, the methacrylation of CS with GMA showed superior reactivity, yielding higher degrees of methacrylation.^257^ The synthesized methacrylated CS can be further cross-linked by photochemical activation of the double bond, allowing the combination of methacrylated CS with different synthetic and natural polymers with application in drug-delivery, cell-encapsulation, or tissue-engineering.^259–261^

**Scheme 15:**
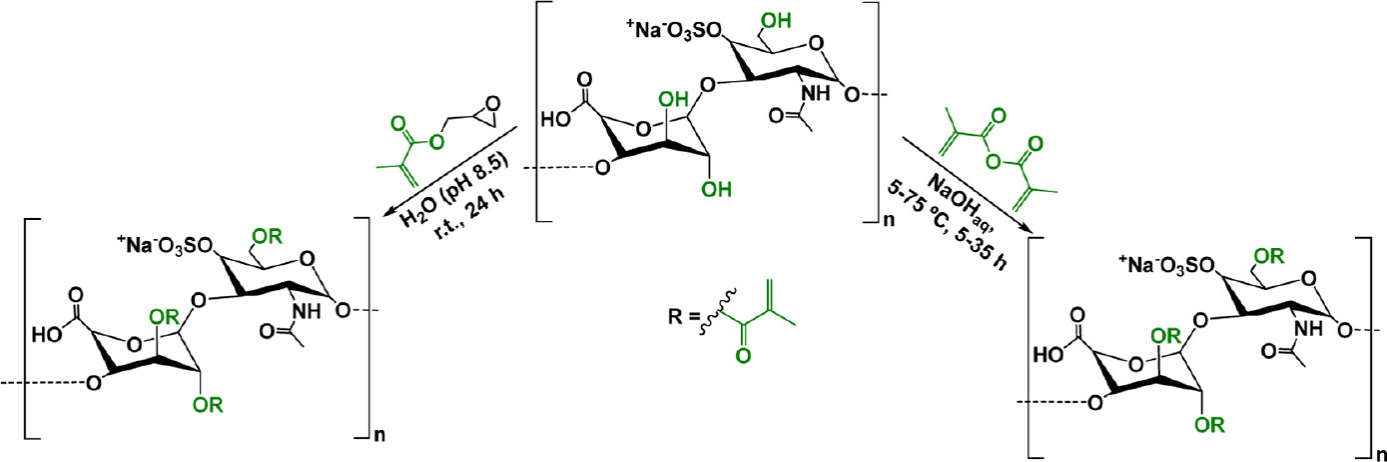
Methacrylation of CS with Glycidyl Methacrylate (Left) and Methacrylic Anhydride (Right)

#### 3.7.2 Biomedical Applications

##### 3.7.2.1 Native Polysaccharide

Fucoidan, a sulfated polysaccharide found in marine algae, exhibits various biological activities, including anticoagulant and anti-inflammatory properties, making it a promising candidate for the development of biomaterials such as hydrogels, capsules, and coacervates.^239–242^ Notably, many of these biomaterials do not require complex chemical modifications to achieve their desired functionalities. Instead, the physical cross-linking of fucoidan with other macromolecules allows the development of effective biomaterials for a range of biomedical applications, including cell culture,^239^ cell-based immunotherapies,^241^ controlled drug delivery systems,^242^ and tissue regeneration.^240^

Encapsulating pancreatic islets within ALG hydrogels presents a promising approach for treating type 1 diabetes. Nevertheless, the effectiveness of this strategy is hindered by inflammation and hypoxia-induced oxidative stress, which arise from both the encapsulation process and the hydrogel material itself. These factors contribute to reduced insulin secretion and compromise both short- and long-term cell viability. To address this issue, a recent study reported that incorporating fucoidan into ALG microcapsules, using a droplet generator and BaCl_2_ as the gelling solution, significantly reduced oxidative stress in primary rat islets or beta cells, resulting in increased cell viability and enhanced insulin secretion.^239^ Additionally, it has been reported that fucoidan exhibits a specific binding affinity for Interleukin-2 (IL-2), making it an effective vehicle for IL-2 delivery in cancer treatment. To accomplish this, the authors developed a fucoidan-based complex coacervate by combining negatively charged fucoidan with positively charged poly-l-lysine (PLL) at physiological pH. Furthermore, to enable localized intratumoral delivery of IL-2, they developed an injectable coacervate-gel composite system by encapsulating the fucoidan-based coacervates within a Pluronic F-127 hydrogel.^241^ While fucoidan has demonstrated potential as a delivery vehicle for IL-2 in cancer therapy, its potential also extends to tissue engineering. Fucoidan has been incorporated into ALG hydrogels to support chondrogenesis in human mesenchymal stem cells (hMSCs), creating a chondro-inductive microenvironment that enhances cartilage-specific marker expression and reduces hypertrophy, a common issue in ALG encapsulation.^240^ As mentioned above, these systems based on physical cross-linking suffer from a high rate of fucoidan diffusion. As a result, a recent study has reported the development of biosynthetic fucoidan hydrogels through covalent cross-linking between methacrylated fucoidan and synthetic methacrylated poly­(vinyl alcohol) (PVA-MA). These hydrogels exhibit high stability, consistent fucoidan retention, enhanced beta cell viability, and improved glucose responsiveness, demonstrating their potential as a suitable and versatile material for 3D cell encapsulation.^243^

##### 3.7.2.2 Methacrylation

The effect of methacrylated CS on the stiffness and chondrogenic differentiation was recently evaluated.^262^ Methacrylated gelatin and methacrylated CS were combined to produce hydrogels with two distinct stiffness values (ca. 8.4 and 33.4 kPa). For the same concentration of methacrylated CS, stiffer hydrogels presented enhanced chondrogenesis of the mesenchymal stem cells. Leveraging the same chemistry, 3D photo-cross-linkable ulvan-derived bioactive scaffolds were produced, showcasing a high capability to induce biomineralization and, consequently, trigger bone regeneration.^244^ The scaffolds were soaked in an alkaline phosphate (ALP) solution to potentiate mineralization. The results indicated that following incubation in a mineralization-inductive medium, the hydrogel combined with ALP exhibited a surface covered with hydroxyapatite crystals, whereas the ALP-free hydrogel showcased no crystal formation on its surface. Interestingly, the hydrogels exhibited distinct crystal morphologies and different Ca/P ratios while varying the concentration of ALP (Figure 8A). Lastly, *in vitro* studies revealed that the scaffolds hold great promise to induce osteogenic differentiation in MC3T3-E1 cells (Figure 8B).

**Figure 8:**
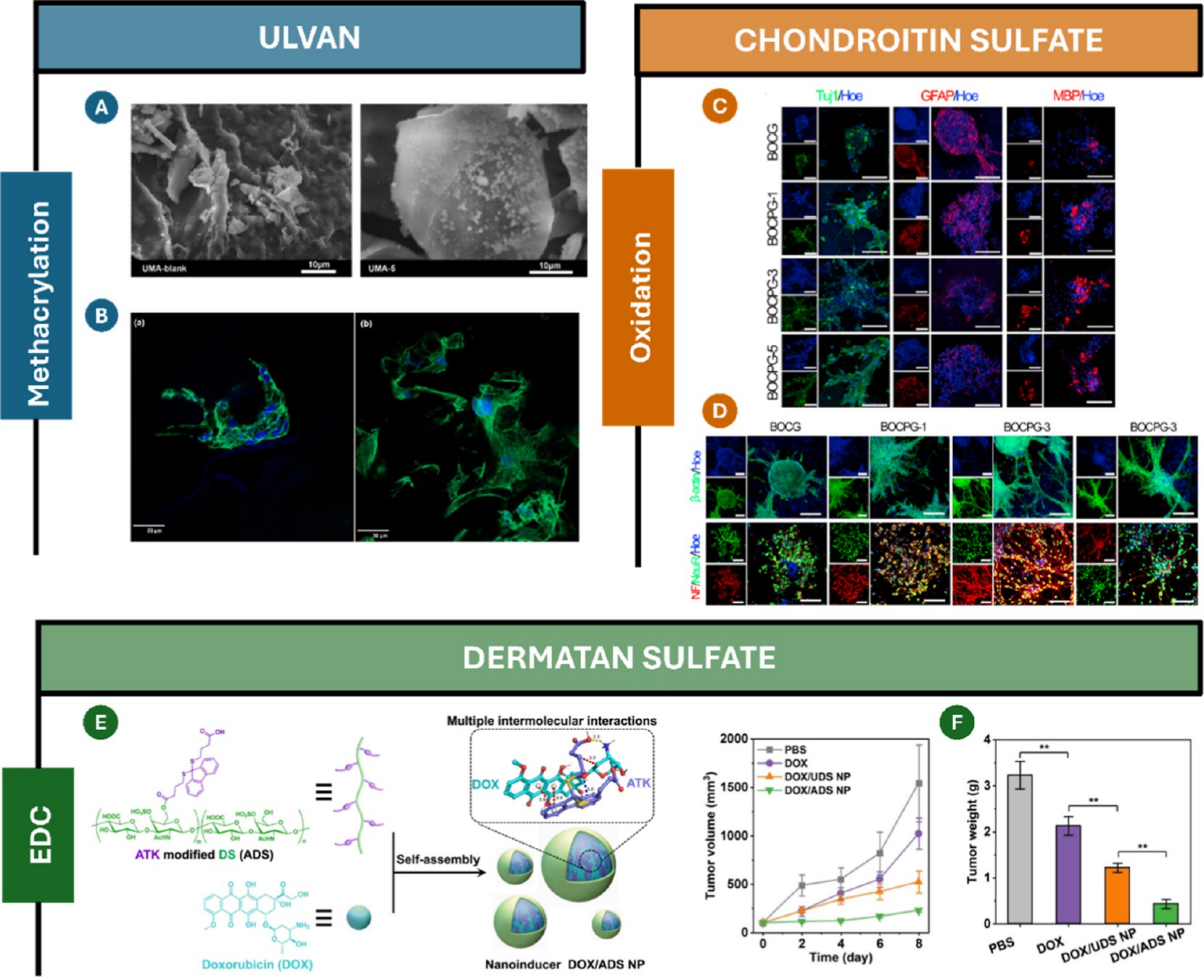
(A) SEM images of methacrylated ulvan-based scaffolds; left, without (blank) and right, with 5 mg/mL of ALP; (B) DAPI/Phalloidin staining of MC3T3 cells seeded on the scaffolds after 14 days of culture. Reproduced with permission from.^244^ Copyright 2014 American Chemical Society; neural stem cell (NSC) differentiation (C) and axon outgrowth (D) on BOCP-Gelatin hydrogels. Reproduced with permission from.^261^ Copyright 2022 ScienceDirect; (E) design of a nanoinducer based on the coassembly of DOX and ADS through multiple intermolecular interactions; (F) tumor volume evaluation over time (left) and average tumor weight at the end of treatment (right). Reprinted with permission from Elsevier.^269,270,271,272,273,274,275,276,277,278,279,280,281^

##### 3.7.2.3 Oxidation

CS is ubiquitous in the ECM of the central nervous system (CNS), playing an important role in axonal guidance during embryonic CNS formation.^263^ In this regard, an injectable, electroconductive and self-healable hydrogel was produced by mixing borax-functionalized oxidized CS (BOC), BOC-doped polypyrrole (BOCP), and gelatin.^261^ The hydrogel was formed by combining covalent and noncovalent interactions. Nonetheless, it is worth noting that the preoxidation of CS allowed its further functionalization with borax moieties that, ultimately, undergo Schiff base reactions. It was found that the hydrogel denoted electroconductive behavior (4.49 mS/cm) due to the presence of polypyrrole units. Moreover, when injected into an injury inflicted in the spinal cord, the hydrogel promoted neuronal differentiation, enhanced axon outgrowth, and inhibited astrocyte differentiation, being highly promising for neuronal regeneration (Figure 8C,D). The impact of ulvan oxidation on its anticoagulant activity has been also evaluated,^264^ revealing a distinct reduction in clot formation time for the oxidized ulvan derivatives. Nonetheless, it was also demonstrated that their anticoagulant activity dependence is tied to the presence of sulfate groups in the native polymer.

##### 3.7.2.4 Carbodiimide-Mediated Reactions

CS-based hydrogels have demonstrated to promote bone and cartilage regeneration.^265–267^ Tyramine was conjugated to both CS and HA, yielding a hydrogel by oxidative coupling of the grafted tyramine moieties via hydrogen peroxide (H_2_O_2_) and horseradish peroxidase (HRP).^250^ The incorporation of both components resulted in a hydrogel with favorable stiffness and elasticity. In fact, when compressed to up to 75% of its height, the hydrogel still exhibited a good recovery of its original shape. In addition, despite the higher toughness achieved with increasing concentrations of both polysaccharides, the hydrogels lost their ability to recover after experiencing significant deformation (compared with less concentrated hydrogels). Even though H_2_O_2_ had a negative effect on cell viability, no statistically significant differences were found when compared to the control group (without H_2_O_2_).

To mitigate side effects and enhance the specificity of cancer therapy, it is crucial to develop innovative delivery systems.^268^ Zhang et al.^269^ designed a nanoparticle assembly system (nanoinducer) based on DS grafted with aromatic thioketal (ADS) via an EDC coupling reaction. This system enables self-assembly through supramolecular interactions with doxorubicin (DOX/ADS NP; Figure 8E). It was found that, upon treatment with this system, melanoma B16F10 cells expressed higher levels of immunogenic cell death markers, thus triggering the immune response. Actually, *in vivo* studies demonstrated that mice treated with the proposed drug delivery system exhibited slower tumor growth and smaller tumor weight, indicating a strong antitumor efficacy (Figure 8F).

## 4 Conclusions

This review systematically overviews the most widely used cationic, anionic, and neutral marine-origin polysaccharides, their main renewable sources and extraction methods, and their physicochemical, structural, and biological properties. Particular emphasis is put on the diversity of chemical modification routes for extending the functionality of marine polysaccharides, which are a valuable alternative to petroleum-derived polymers and for assigning them with improved physicochemical and biological properties to address specific biomedical applications. Those include bioactivity, stimuli-responsiveness, dynamic and bioinstructive behavior, and enhanced mechanical properties. In addition, this review covers several nano- and microtechnologies to enable the processing of functionalized polysaccharides into a diverse set of high added-value tunable biomaterials, denoting emerging properties and multiple functionalities—spanning from nano-/microparticles, capsules, nanofibers, multilayered thin films, and free-standing membranes to hydrogels or cryogels. Moreover, the intrinsic properties of marine polysaccharides (e.g., charge and structural composition) play a crucial role in defining their performance and should not be overlooked. For instance, the structural units of LAM can be leveraged to create enzymatically controlled self-feeding hydrogels,^282,283^ whereas ionizable groups enable the electrostatic assembly of ECM-mimetic bioactive platforms^284,285^ or support the formation of robust double-network hydrogels.^286^ Such advanced polymeric biomaterials hold great promise for antimicrobial purposes, drug delivery, tissue engineering, regenerative medicine, and *in vitro* disease modeling.

## 5 Future Perspectives

Despite the advancements achieved to date in terms of extraction procedures, chemical modification strategies, and developed renewable marine-origin polysaccharide-based biomaterials, there is still much to be explored in taking full advantage of the versatility and potential imparted by the marine polysaccharides toward bioapplications.

Although marine polysaccharides have remarkable potential, several intrinsic limitations still hinder their broader application as advanced and sustainable biomaterials. For instance, the unsuitable mechanical properties or the lack of bioactive features denoted by most marine polysaccharides limit their potential to be translated into advanced and sustainable biomaterials, i.e., materials with a lower carbon footprint, for use in a wide array of biomedical applications. These challenges can be circumvented by their complexation or chemical functionalization with other biocompatible polymeric materials or cell adhesive peptide sequences (e.g., RDG or IKVAV), aiming to develop smart biomaterials with enhanced tunability and functional versatility to trigger the desired cell responses. In fact, the possibility of developing such hybrid polysaccharide-based derivatives, denoting improved mechanical and biological properties and multifunctionalities, opens up new avenues and perspectives in the biomedical field, in particular in modular tissue engineering and regenerative medicine strategies. However, the chemical functionalization of marine polysaccharides with peptide sequences and the scale-up of the resulting biomaterial derivatives are challenging mainly due to the high cost, which limits the scalable manufacturing and industrial projection of the developed biomaterial derivatives and their mass production toward commercialization. In this regard, the synthesis of bioactive analogue molecules that would imply a sustainable cost-benefit potential compatible with the industrial scale-up might open new perspectives. In addition, the lack of efficient, cost-effective, eco-friendly, and sustainable standardized industrial-scale extraction methodologies that would simultaneously reduce energy consumption and environmental impact, and adequate purification methodologies hamper their translation from the lab bench into industrial settings toward commercialization. Furthermore, the translation of such biomaterials into the clinics faces additional bottlenecks in terms of obtaining reproducible medical grade polysaccharides from raw heterogeneous natural sources, purity level, batch-to-batch variability, or stability. Furthermore, the stringent quality control requirements and very demanding regulatory hurdles, mainly due to the safety requirements, need for appropriate sterilization procedures, and difficulty in standardization owing to the structural complexity of marine polysaccharides, delay the translation and realization of the full potential of the marine polysaccharide-based biomaterials. Moreover, the structural and biological complexity imparted by natural polymers, and the need for fully understanding the mechanisms of action, reaction pathways, structural-property-function relationships and effects on the human body, hinder predictable performance and delay their successful translation into ready-to-use biomaterials to be applied in the clinics. As such, strong emphasis should be placed on the comprehensive long-term evaluation of their structure, biochemical, and biological properties and extensive *in vivo* testing to boost their potential translation into clinical settings. The authors believe that the possible prediction of the polysaccharides’ properties and performance by computational simulations, together with experimental data, will undeniably contribute to clarifying several of these questions and open new opportunities for real-world applications and future commercialization in a faster and cheaper way. Moreover, the increased *trans*-disciplinary and sectorial collaboration between academia, biotechnological companies, and regulatory bodies in the upcoming decades will be of paramount importance in addressing most of these bottlenecks and enable meaningful progress toward cost-effective, scalable, and eco-friendly extraction technologies. Such collaborative efforts will be crucial in fully identifying the untapped potential of marine polysaccharides and opportunities to accelerate the pace on their widespread industrial and clinical translation and full market potential, opening new avenues for their sustainable, safe, and personalized use in biomedicine and healthcare to improve human health.
